# MAFF regulates ferroptotic sensitivity through iron homeostasis and fatty acid synthesis

**DOI:** 10.1038/s41419-026-08885-w

**Published:** 2026-05-28

**Authors:** Nuria Vilaplana Lopera, Jiyoung Kim, Pei Ju Lee, Maxime Dubail, Shuhei Aramaki, Maria Jerome, Adela Valentova, Tianxu Hou, Haowen Jiang, Stephano Spano Mello, Eun-Woo Lee, Christian Babbs, Theo Rich, Yu Xu, Yanyan Jiang, Charles Fouillade, Mitsutoshi Setou, Jiangbin Ye, Eui Jung Moon

**Affiliations:** 1 Department of Oncology, University of Oxford, Oxford, UK; 2 Department of Radiation Oncology, Yonsei University College of Medicine, Seoul, Republic of Korea; 3 Department of Cellular and Molecular Anatomy, Hamamatsu University School of Medicine, Hamamatsu, Japan; 4 Photonic Quantum Therapeutics Laboratory, Institute of Photonics Medicine, Hamamatsu University School of Medicine, Hamamatsu, Japan; 5 Department of Radiation Oncology, Hamamatsu University School of Medicine, Hamamatsu, Japan; 6 Department of Radiation Oncology, Stanford University, Stanford, CA USA; 7 Department of Biomedical Genetics, University of Rochester, Rochester, NY USA; 8 Metabolic Regulation Research Center, Korea Research Institute of Bioscience and Biotechnology (KRIBB), Daejeon, Korea; 9 School of Pharmacy, Sungkyunkwan University, Suwon, Republic of Korea; 10 Department of Functional Genomics, University of Science and Technology (UST), Daejeon, Republic of Korea; 11 Genome Engineering Facility, MRC Weatherall Institute of Molecular Medicine, John Radcliffe Hospital, University of Oxford, Oxford, UK; 12 Institut Curie, Inserm U1353-CNRS UMR9029, University Paris-Saclay, PSL Research University, Centre Universitaire, Cedex, France; 13 International Mass Imaging and Spatial Omics Center, Institute of Photonics Medicine, Hamamatsu University School of Medicine, Hamamatsu, Japan; 14 International Center for Aging and Cancer, Hainan Medical University, Haikou, China

**Keywords:** Breast cancer, Cancer microenvironment

## Abstract

Ferroptosis is an iron-dependent form of regulated cell death driven by lipid peroxidation. In this study, we identify the transcription factor *v*-maf musculoaponeurotic fibrosarcoma oncogene homolog F (MAFF) as a key regulator that promotes breast cancer progression while simultaneously sensitizing tumor cells to ferroptosis. Integrated RNA-sequencing and chromatin immunoprecipitation (ChIP)-sequencing revealed that MAFF directly regulates genes involved in iron and fatty acid metabolism. MAFF enhances intracellular iron availability by transactivating *SLC11A2* and *NCOA4*, expanding the labile iron pool essential for ferroptotic signaling. In parallel, MAFF represses *CPT2*, *FASN*, and *SCD1*, reprogramming lipid metabolism to favor the accumulation of polyunsaturated fatty acids (PUFAs) over monounsaturated fatty acids (MUFAs), thereby increasing lipid peroxidation susceptibility. These alterations reduce triglyceride (TG) levels and lipid droplet (LD) formation following ferroptosis induction. Functionally, MAFF-mediated repression of *CPT2* diminishes fatty acid oxidation and enhances tumor invasion, underscoring a dual role whereby MAFF drives tumor progression yet primes cells for ferroptotic death. Collectively, our study identifies MAFF as a transcriptional switch linking iron homeostasis and lipid metabolism to ferroptotic vulnerability, highlighting its potential as a biomarker and therapeutic target in basal-like breast cancer.

## Introduction

Ferroptosis is a form of regulated cell death that depends on iron availability and the accumulation of lipid peroxides [1, 2]. Iron turnover from ferric iron to ferrous iron drives the Fenton reaction, which produces free radicals and initiates lipid peroxidation specifically of polyunsaturated fatty acids (PUFAs) [3]. In the presence of iron and oxygen, the chain reaction of lipid peroxidation will continue and eventually lead to cell death from the accumulation of lipid reactive oxygen species (ROS). However, cells have antioxidant defense mechanisms specifically against lipid ROS, primarily through the action of the glutathione peroxidase 4 (GPX4) enzyme. GPX4 reduces lipid hydroperoxides into non-toxic lipid alcohols using glutathione (GSH) as a cofactor; hence, its action is dependent on glutathione synthesis from cysteine, which is imported as cystine [1, 4]. System Xc^-^, a cystine/glutamate antiporter composed of the transporter subunit SLC7A11 and the regulatory subunit SLC3A2, is crucial for cystine uptake [5]. Therefore, targeting SLC7A11 or GPX4 results in ferroptosis.

A growing body of evidence indicates that cancer subtypes associated with high-grade malignancy and therapeutic resistance are frequently sensitive to ferroptosis induction [1, 6–11]. Tumors exhibiting mesenchymal-like features, acquired drug resistance, or specific genetic alterations have evolved mechanisms to adapt to diverse cellular stresses. Paradoxically, these same adaptations appear to impose a unique vulnerability to ferroptosis that remains mechanistically unresolved.

In our previous study, we identified *v*-maf musculoaponeurotic fibrosarcoma oncogene homolog F (MAFF) as a hypoxia target gene significantly promoting tumor cell invasion and metastasis [12]. MAFF is a bZIP transcription factor that belongs to the small MAF family, including MAFF, MAFK and MAFG [13, 14]. Small MAF proteins lack transactivation domains and form homodimers or heterodimers with the CNC family (NF-E2, NRF1, NRF2, NRF3) and BACH family proteins (BACH1 and BACH2) [15]. Therefore, the functional role of small MAF proteins is critically determined both by their own expression and by the availability of binding partners. While the antagonistic roles of NRF2 and BACH1 in ferroptosis regulation are well-characterized [16–19], the specific contribution of MAFF to this pathway, beyond its function as an indispensable binding partner, has not been fully elucidated.

In this study, we demonstrate that MAFF plays an essential role by activating genes controlling iron homeostasis to expand the labile iron pool, while simultaneously repressing fatty acid metabolic genes to establish a lipid profile prone to peroxidation. This work establishes MAFF as a key transcriptional switch that mechanistically links iron and lipid metabolism to ferroptotic susceptibility.

## Materials and methods

### Cell lines

MDA-MB-231 and Hs578T cell lines were purchased from ATCC. BT549 were kindly provided by Professor Ester Hammond at University of Oxford. Cell lines were maintained at 37°C in a humidified incubator at 5% CO_2_. Pairs of MDA-MB-231 shSCR and shMAFF, and MDA-MB-231 Vector and MAFF overexpression (OE) cells were prepared as previously described [12]. CPT2-overexpressing cells were generated by infection with a lentiviral CPT2 vector. Human CPT2 cDNA (Horizon Discovery, MHS6278-202827588; Clone ID: 3346025) was cloned into the pLX304 vector containing a V5 tag and a blasticidin resistance cassette by the Genome Engineering Facility at the Weatherall Institute of Molecular Medicine, University of Oxford. Lentiviral particles were produced using the Dharmacon™ Trans-Lentiviral Packaging Kit (TLP5917) according to the manufacturer’s protocol. The collected viral supernatant was filtered through a 0.4 µm PVDF filter and used to infect MDA-MB-231 cells in the presence of 5 µg/mL polybrene. Forty-eight hours post-infection, cells were selected with 10 µg/mL blasticidin. Selection was continued for two weeks. All cell lines were cultured in Dulbecco’s Modified Eagle Medium supplemented with 10% FBS (F7524, Sigma Aldrich) and 1% antibiotic/antimycotic (15240-062, Gibco). Cells were routinely tested for mycoplasma.

### Reagents

Erastin (B1524, APExBIO) was dissolved in DMSO to prepare a 5 mM stock concentration. RSL-3 (S8155, Selleckchem) and Ferrostatin-1 (Ferr-1, ab146169-5mg, Abcam) were dissolved in DMSO to prepare a 1 mM and 10 mM stock solutions, respectively. Oleic acid (O1008, Sigma Aldrich) and linoleic acid (62240, Sigma Aldrich) were prepared fresh as a 350 mM stock solutions in DMSO. Ammonium iron (II) sulfate (09719, Merck) was dissolved in deionized water to prepare a 100 mM stock solution. All these reagents were diluted in cell culture media to specified concentrations.

### Cell survival assay

Cell survival was determined using the CellTiter-Blue® assay or by DAPI-based cell counting performed on the Celigo imaging system. Five thousand cells were seeded per well in 96-well plates and incubated for 24 h, followed by treatment with ferroptosis inducers or inhibitors for 24 h. Cells were then washed with PBS. For the CellTiter-Blue® assay, CellTiter-Blue® reagent (Promega) diluted 1:5 in PBS was added according to the manufacturer’s instructions and incubated for 1 h at 37 °C as previously described [20]. Fluorescence (Ex 544 nm/Em 590 nm) was measured using a POLARstar Omega plate reader (BMG Labtech). For DAPI staining, cells were fixed with 4% formaldehyde solution (Thermo Fisher Scientific) for 7 min, washed with PBS, and stained with 1 μg/ml DAPI. DAPI-based cell counts were analyzed using the built-in software of the Celigo Image Cytometer (Nexcelom).

### PI staining

Propidium iodide (PI) staining was used to quantify the loss of plasma membrane integrity as a measure of cell death. Cell culture supernatant with floating cells as well as attached cells were collected and incubated with 5 µg/ml PI in PBS for 10 min at room temperature. PI fluorescence was measured in the PE channel on a CytoFLEX flow cytometer (Beckman Coulter). Data were analyzed using FlowJo. Unstained samples were used to define negative populations, and PI-positive cells were considered as dead cells.

### Lipid peroxidation and lipid droplets measurements

Lipid peroxidation was assessed using the BODIPY 581/591 C11 (D3861, Invitrogen) as previously described [21]. Mitochondrial lipid peroxidation and lipid droplet content were determined using MitoPerOx (ab146820, Abcam) and the BODIPY 493/503 (D3922, Invitrogen) probes according to the manufacturer’s instructions. Briefly, 150,000 cells were plated in 6-well plates and incubated for 24 h. Cells were then treated with vehicle or ferroptosis modulating compounds at the specified concentrations for another 24 h, washed with PBS, and incubated with 2 µM BODIPY 581/591 C11 (lipid peroxidation), 5 µM of MitoPerOx or 5 µM BODIPY 493/503 (lipid droplets) in PBS for 30 min at 37 °C. For flow cytometry, cells were detached and washed with PBS before FITC fluorescence intensity (oxidized BODIPY C11 and MitoPerOx emission or BODIPY 493/503) was measured using a CytoFLEX (Beckman Coulter) flow cytometer. FlowJo software was used to assess the geometric mean of the intensity of oxidized BODIPY 581/591 C11 and MitoPerOx, or the intensity of BODIPY 493/503.

For Celigo analysis, cells in 96-well plates prepared as described above were stained with 2 µM BODIPY 581/591 C11 in PBS for 30 min at 37 °C, fixed with 4% formaldehyde (Thermo Fisher) for 7 min, washed with PBS, and subsequently stained with 1 µg/ml DAPI. BODIPY C11 fluorescence (Ex 483 nm/Em 536 nm) and DAPI (Ex 377 nm/Em 447 nm) were directly imaged after the incubation using the Celigo Image Cytometer (Nexcelom). Cell counts based on DAPI staining and the fluorescence intensity of BODIPY C11 were analyzed with the built-in software of the Celigo Image Cytometer.

### siRNA transfection

ONTARGETplus SMARTpool siRNA targeting *MAFF*, *SLC11A2*, *NCOA4*, *CPT2*, *FASN*, and *SCD1* (Horizon Discovery) were transfected into cells according to the manufacturer’s instructions. Briefly, 2 × 10⁵ or 5000 cells were seeded in 6-well or 96-well plates, respectively. siRNAs were complexed with Lipofectamine RNAiMAX (13778150, Invitrogen) using Opti-MEM (31985070, Gibco) media and incubated at room temperature for 20 min. The siRNA-lipid complexes were added onto washed cells in antibiotic-free DMEM supplemented with 10% FBS at a final concentration of 100 nM. Cells were incubated for 16 h, and the medium was replaced with complete medium. Treatments were performed 24–48 h after siRNA transfection.

### RNA sequencing and analysis

RNA samples were extracted from MDA-MB-231 control (shSCR) and MAFF knockdown (shMAFF) cells. Briefly, RNA was extracted using the RNeasy Mini Kit (74104, Qiagen) following the manufacturer’s protocol. Library preparation, sequencing (paired-end 150 bp sequencing, 20 million reads), and bioinformatic analysis were performed by Novogene Co. (Cambridge, UK) using the Illumina NovaSeq platform. Genes significantly upregulated or downregulated upon MAFF knockdown (≥1.3-fold change) were identified, and overlapping genes with our previous ChIP-sequencing data [12] were further refined. These genes were then analyzed using Enrichr, focusing on Kyoto Encyclopedia of Genes and Genomes (KEGG) pathway analysis [22–24]. Enrichment plots were subsequently generated with the SRplot tool to visualize the pathway enrichment results [25]. The ferroptosis-related gene list we identified was further analyzed and mapped using KEGG Mapper [26, 27]. The Gene Set Enrichment Analysis (GSEA) was performed on the combined set of upregulated and downregulated genes using gene sets from the Molecular Signatures Database (MSigDB v2024.1.Hs) [28]. Raw gene count data were normalized to transcripts per million (TPM) prior to GSEA analysis. Analyses were conducted using GSEA software (v4.3.3) from the Broad Institute. Gene sets with nominal *P*-value < 0.05 and False Discovery Rate (FDR) < 25% were considered significantly enriched. Additionally, the heatmap was generated using Python (3.11.8).

### Intracellular iron measurements

Intracellular iron levels were measured using the FerroOrange (F374, Dojindo) probe according to the manufacturer’s instructions. Briefly, 5000 cells were seeded per well in a 96-well plate and incubated for 24 h. Cells were then treated with erastin or 50 µM ammonium iron sulfate for an additional 24 h, washed with PBS, and incubated with 1 µM FerroOrange in serum-free, phenol red–free medium (31053028, Gibco) for 30 min at 37 °C. Fluorescence (Ex 531/40 nm/Em 629/53 nm) was directly imaged after the incubation using the Celigo Image Cytometer (Nexcelom).

### CTRP analysis

The Cancer Therapeutics Response Portal (CTRP) was used to correlate expression levels of *SLC11A2*, *NCOA4*, *CPT2,* *FASN*, and *SCD1* with drug sensitivity (area under the dose-response curve, AUC) in breast cancer cell lines. The resulting correlation plots were exported directly from the CTRP, and ferroptosis-inducing compounds were labeled.

### Lipidomic analysis

To assess MAFF-specific alterations in lipid profiles, lipidomic analysis was performed on MDA-MB-231 cells with or without MAFF expression (shSCR and shMAFF). Cells were seeded in 10-cm dishes at a density of 1 × 10⁶ cells per dish in biological triplicate prior to lipid extraction and analysis. The following day, cells at approximately 80% confluence were scraped into 1.5 mL of ice-cold methanol and transferred to glass tubes. Lipids were extracted by adding 5 mL methyl tert-butyl ether (MTBE), vortexing, and incubating for 1 h at room temperature with shaking, followed by addition of 1.25 mL water and a further 10 min incubation. After centrifugation (1000 × *g*, 10 min), the upper organic phase was collected and dried under nitrogen. Dried extracts were reconstituted in methanol:toluene (9:1, v/v) containing 10 mM ammonium acetate for LC–MS analysis.

Samples were analyzed on an Agilent 1290 Infinity II UHPLC coupled to a 6546 Q-TOF mass spectrometer equipped with a C18 reverse-phase column (2.1 × 100 mm, 1.7 µm). Mobile phase A was water/acetonitrile (60:40, v/v) with 10 mM ammonium formate and 0.1% formic acid, and mobile phase B was isopropanol/acetonitrile (90:10, v/v) with the same additives. The gradient was 15–100% B over 15 min at 0.4 mL/min, followed by 3 min re-equilibration at 15% B. The column temperature was 55 °C and the autosampler 4 °C. The Q-TOF was operated in both positive and negative ESI modes with a capillary voltage of ±3.5 kV, fragmentor 120 V, drying gas 250 °C (12 L/min), sheath gas 300 °C (11 L/min), and nebulizer pressure 35 psi.

A pooled sample was repeatedly analyzed in iterative auto-MS/MS mode to expand MS² coverage. The combined data were processed with Agilent Lipid Annotator to construct an MS¹ library, which was then used to annotate individual MS¹-only sample runs. Lipid features were aligned and quantified from MS¹ peak areas, with annotation supported by accurate mass, retention time, and library matches in both polarities. Final data were normalized to protein concentrations of each sample. A heatmap was generated using Morpheus (https://software.broadinstitute.org/morpheus), displaying lipid classes that showed significant changes (*p* < 0.05) as determined by Student’s *t*-test.

To determine the impact of CPT2 overexpression on lipid composition, MDA-MB-231 cells stably expressing CPT2 or the corresponding empty vector control were seeded in 10-cm dishes at a density of 4 × 10⁶ cells per dish, with five biological replicates per group. The following day, cells were treated with DMSO or 2.5 µM erastin for 24 h. Cells were then washed with PBS, scraped, collected, and counted, and cell pellets were snap-frozen in liquid nitrogen. Lipid extraction and subsequent lipidomic analyses were performed by Preppers Inc. (Shizuoka, Japan). To prevent artifactual lipid peroxidation during the extraction procedure, butylated hydroxytoluene (BHT) was added to methanol at a final concentration of 0.01% (w/v). Whole cell lipids were extracted by the Bligh and Dyer method with slight modifications for LC-MS/MS analysis. Briefly, cell pellets were resuspended in methanol containing 0.01% BHT, with the volume adjusted according to the number of cells. Samples were homogenized in glass tubes and subjected to sonication at high power (5 cycles of 20 s each). After additional homogenization, 2 mL of each sample homogenate was transferred to a clean glass tube. Subsequently, 800 µL of ultrapure water was added, followed by 1 mL of chloroform containing internal standard PC(12:0_12:0) at a concentration of 10 ng/μL. Samples were vortexed thoroughly and allowed to equilibrate for 10 min. An additional 1 mL of chloroform and 1 ml of 0.28 M acetate were then added. The mixture was vortexed again and centrifuged at 1500 rpm for 15 min at 4 °C to ensure clear phase separation. After centrifugation, 1.6 mL of the lower organic phase containing the extracted lipids was carefully collected. The organic solvent was evaporated under desiccation for 1 h. Dried lipid extracts were stored at −80 °C until mass spectrometry analysis. Before performing the LC-MS/MS analysis, the dried lipid sample was reconstituted in 100 μL of pure methanol containing 0.01% BHT and then transferred into LC-MS insert vials.

Lipidomic analyses were performed using a Q Exactive Hybrid Quadrupole-Orbitrap mass spectrometer (Thermo Fisher Scientific, USA) coupled to an Ultimate 3000 ultra-high-performance liquid chromatography (UHPLC) system (Thermo Fisher Scientific). Lipid species were ionized using an electrospray ionization (ESI) source operating in both positive and negative ion modes. Chromatographic separation was achieved on a hydrophobic Acclaim 120 C18 column (150 × 2.1 mm, 3 µm; Thermo Fisher Scientific). The column temperature was maintained at 50 °C, and the autosampler was kept at 10 °C. The injection volume was 1 µL. The flow rate was set to 0.30 mL/min. The mobile phases consisted of solvent A (water:acetonitrile:methanol, 2:1:1, v/v/v) and solvent B (acetonitrile:isopropanol, 1:9, v/v), both supplemented with 5 mM ammonium formate and 0.1% formic acid. The gradient program was as follows: 20% B at 0 min, increased linearly to 100% B over 50 min, held at 100% B for 10 min, returned to 20% B at 60.1 min, and maintained at 20% B until 70 min for column re-equilibration. A blank injection (pure methanol) was performed prior to each sample run. Mass spectrometry data were acquired using Xcalibur v3.0 software (Thermo Fisher Scientific) in both full MS and data-dependent MS/MS (dd-MS²) modes. For full MS acquisition, parameters were set as follows: *m*/*z* range 220–2000, resolving power 70,000 (FWHM at *m*/*z* 200), automatic gain control (AGC) target 1 × 10⁵, and maximum injection time (IT) 100 ms. For dd-MS² acquisition, the following parameters were applied: resolving power 17,500 (FWHM at *m*/*z* 200), AGC target 1 × 10⁵, maximum IT 80 ms, TopN 5, isolation window 2.0 *m*/*z*, normalized collision energy (NCE) 30 eV, loop count 5, and dynamic exclusion 15 s. Stepped NCE was set to 15% in positive mode and 35% in negative mode. Source conditions were optimized as follows: spray voltage 3.5 kV (positive mode) and 2.5 kV (negative mode); capillary temperature 250 °C; probe heater temperature 350 °C; sheath gas flow rate 50 (arbitrary units); auxiliary gas flow rate 15 (arbitrary units); sweep gas flow rate 0; and S-lens RF level 50.

LC-MS/MS for lipidomic data was analyzed by XcaliberTM (Thermo Fisher) and LipidSearchTM (Thermo Fisher). Lipidomic data were normalized by internal standard [PC 12:0/12:0]. Raw lipidomic data were processed in R (v.4.4) using a standardized pipeline. Lipid species with excessive missing values were excluded using a strict filter (more than two missing values per condition). When multiple features corresponded to the same lipid annotation, only the most intense feature was retained. Intensities were first normalized on the linear scale using the internal standard PC(12:0/12:0) to correct for extraction and analytical variability. Internal standard–normalized values were then normalized using a median-of-ratios (MoR) approach, in which sample-specific size factors were calculated from the median ratio of lipid intensities to lipid-wise geometric means computed across a reference set detected in at least 50% of samples. Normalized data were log2-transformed, and remaining missing values were imputed using a minimum detection (MinDet) approach. A final biochemical filtering step was applied to retain mammalian-relevant lipid species based on lipid class, acyl chain number, chain length, and degree of unsaturation. The resulting dataset was used for all downstream analyses. Differential analysis was performed on IS + MoR–normalized log₂ lipid intensities using linear models with empirical Bayes moderation (limma). Lipids with *p* < 0.05 and |log₂FC| > 0.5 were considered significantly regulated.

### Quantitative RT-PCR

Total mRNA was extracted using TRIzol^TM^ Reagent (15596026, Invitrogen) following manufacturer’s instructions. One μg of mRNA was reverse transcribed into cDNA using an iScript cDNA synthesis kit (1708890, Bio-Rad). Quantitative real-time RT-PCR was performed with an iTaqTM Universal SYBR Green Supermix (1725124, Bio-Rad) using the StepOnePlus Real-Time PCR system (Applied Biosystems). Expression level of each target mRNA (see table below) was determined using the ΔΔCT method and was normalized to 18S expression in the same sample.

**Table:** 

SLC11A2	Forward	AGTATGTCACCGTCAGTATCCC
SLC11A2	Reverse	ATCTGCAATGGTGATGAGAACG
NCOA4	Forward	AGACTGACTCCTGTACCAACTG
NCOA4	Reverse	GCACACCTCCTCTACCTTACAT
CPT2	Forward	TCCTGTCCACGAGCACACTGAG
CPT2	Reverse	AGCATACCCAACACCAAAGCCATC
FASN	Forward	CAAGGACACAGTCACCATCTC
FASN	Reverse	CATGAAGTAGGAGTGGAAGGC
SCD1	Forward	ACAACTACCACCACTCCTTTC
SCD1	Reverse	GGAGACTTTCTTCCGGTCATAG
18S	Forward	GAGGATGAGGTGGAACGTGT
18S	Reverse	AGAAGTGACGCAGCCCTCTA

### Western blot

Cells were washed with PBS, detached from the plate, and briefly centrifuged before extracting protein by adding RIPA lysis buffer supplemented with cOmplete Protease Inhibitor Cocktail (04693116001, Roche) and PhosSTOP Phosphatase Inhibitor Cocktail (04906837001, Roche). Following brief sonication, cells were centrifuged at 8500 × *g* for 10 min at 4 °C, and supernatant was used. Protein concentration was determined by using the Pierce^TM^ BCA protein Assay kit (23225, Thermo Fisher) according to manufacturer’s instructions. 40 μg of protein were prepared using Bolt LDS Sample Buffer (B0007, Thermo Fisher) and Bolt Sample Reducing Agent (B0009, Thermo Fisher). Protein samples were denatured at 95 °C and loaded on a Bolt 4-12% Bis-Tris Plus gels (NW04120BOX, Invitrogen). Precision Plus Protein Kaleidoscope (1610375, Bio-Rad) was included as a molecular weight marker. Samples were run in MES SDS Running Buffer (B0002, Invitrogen) at 150 V for 1 h. The Trans-Blot Turbo Transfer buffer (10026938, Bio-Rad) and Trans Blot Turbo Transfer system were used to perform semi-dry transfer using Immun-Blot PVDF membranes. After the transfer, the membranes were blocked in a 5% BSA solution in TBS with 1% Tween-20 (TBST) for an hour. Membranes were then incubated with primary antibodies overnight at 4 °C (see table below). Membranes were washed with TBST and incubated with anti-rabbit, anti-mouse, or anti-goat secondary antibodies (1:5000) or β-actin HRP (sc47778, Santa Cruz, 1:5000) for 1 h. Clarity Western ECL Substrate (1705060, Bio-Rad) was used for membrane development, and the protein bands visualized in a Bio-Rad ChemiDoc system.

**Table:** 

MAFF	M8194	Sigma	1:1000
DMT1	ab55735	Abcam	1:1000
NCOA4	A302-271A	Bethyl	1:1000
CPT2	26555-1-AP	Proteintech	1:1000
FASN	ab128870	Abcam	1:1000
SCD1	28678-1-AP	Proteintech	1:1000
PLIN3	HPA006427	Atlas Antibodies	1:1000

### Invasion assay

Invasion assays were performed as previously described [12]. Briefly, Corning® BioCoat™ Matrigel® Invasion Chambers (#354480, Corning) were used according to the manufacturer’s instructions. Chambers were pre-incubated with FBS- and antibiotic-free DMEM for 2 h in a humidified incubator at 37 °C with 5% CO₂. Cell suspensions (2.5 × 10⁴ cells in 500 µl of FBS- and antibiotic-free DMEM) were seeded into the upper chambers. The lower chambers were filled with 750 µl of DMEM supplemented with FBS and antibiotics. After overnight incubation, non-invading cells on the upper membrane surface were gently removed using cotton swabs. Invading cells on the lower membrane surface were stained with 1 μg/ml DAPI followed by a three-step staining kit (#3313, Epredia™ Richard-Allan Scientific™). Images were acquired using an EVOS imaging system, and three regions of interest were identified and counted per insert. Each experiment was performed in triplicate with two technical replicates. Invading cell numbers were averaged and normalized to the corresponding control group for each independent experiment.

### Mouse studies

All animal experiments were performed according to the guidelines of the United Kingdom Home Office and the University of Oxford under the project license PP4558762. Female athymic nude (Crl:NU (NCr)-*Foxn1*^*nu*^, 6–8 weeks) mice were purchased from Charles River Laboratories and housed in individually ventilated cages containing no more than six mice per cage, in a 12/12-h light/dark cycle. One million MDA-MB-231 shSCR or shMAFF cells were suspended in 25 µl of PBS and 25 µl of Matrigel (Corning 354230) and inoculated into the mammary fat pad subcutaneously. Tumor growth was measured three times a week using calipers. When the tumor volume reached 100 mm^3^, mice were randomized and injected intraperitoneally with 3% DMSO or 50 mg/kg imidazole ketone erastin (IKE) every two days for two weeks. Mice were sacrificed when tumor size reached 800 mm^3^ or 5 days after the last treatment. For tissue processing, tumors were excised, snap frozen in liquid nitrogen and kept at −80 °C. Frozen tumor tissues were cryosectioned into 6 µm sections that were kept at −80 °C until further processing.

### Immunohistochemistry

Frozen tissue sections were air dried for 10 min prior to fixation with 4% paraformaldehyde for 15 min at room temperature. Slides were then washed with PBS three times prior to blocking.

For paraffin-embedded tissue microarrays (TMAs, BR881, US Biomax) or mouse tumor tissues, slides were incubated at 65 °C for 30 min and deparaffinized by immersing in Histo-Clear (National Diagnostics, HS-200) twice for 3 min followed by rehydration in 100%, 70%, and 50% ethanol for 3 min each. Slides were washed with deionized water for 5 min and antigen retrieval was performed in Tris-EDTA buffer (pH = 9) by heating to 110 °C in a pressure cooker for 3 min. The slides were cooled at room temperature for 20 min prior to blocking.

For blocking, slides were incubated with Dual Endogenous Enzyme Block (S2003, Agilent Technologies) for 20 min. The slides were blocked with 10% goat serum for 30 min and incubated with anti-4-HNE antibody (BS-6313R, Thermo Fisher, 1:100), anti-MAFF antibody (GTX120264, GeneTex, 1:400), or anti-CPT2 antibody (26555-1-AP, Proteintech, 1:600) diluted with antibody diluent (ab64211, Abcam) at 4 °C overnight. After washing three times with PBS/0.05% Tween-20, the secondary anti-rabbit antibody with HRP-labeled polymer was added to slides (K4003, Agilent Technologies) and incubated at room temperature for 20 min. Slides were then washed three times with PBS/0.05% Tween-20 and incubated with DAB (3,3′-Diaminobenzidine) (K3468, Agilent Technologies) to visualize the HRP signals. The slides were counterstained with hematoxylin (1.09249.0500, Sigma Aldrich) and mounted with aqueous mounting medium (1.08562.0050, Merck). Slides were imaged with an Aperio Scanscope CS digital pathology scanner (Leica Biosystems Imaging, Inc.) at 20× magnification. For CPT2 and 4-HNE staining, three representative fields were selected per slide at 10x magnification. DAB staining intensity was analyzed using FIJI (Image-J-based open-source software) [29]. Positively stained areas were extracted using color deconvolution, with the brightness and contrast optimized for each set of images, and the values were kept consistent across all images from the same set. Positively stained areas were converted into binary images, and the integrated density values were calculated. The three representative field results were averaged and normalized to shSCR control. Nuclear MAFF staining was quantified using QuPath software (0.6.0). For TMA slides, tumor regions within each core were annotated following TMA dearraying. For mouse tumor sections, three representative fields per slide were selected at 10× magnification for analysis. We utilized the built-in “Positive cell detection” workflow to segment cells and measure the mean nuclear DAB intensity after stain deconvolution. A threshold for the nuclear DAB signal was set to classify individual cells as positive. The final quantification for each core was determined by calculating the percentage of MAFF-positive tumor cells.

### Patient data

Patient survival z-scores for MAFF and CPT2 expression were obtained from PREdiction of Clinical Outcomes from Genomic Profiles (PRECOG) [30] and visualized. To compare prognostic associations across independent data sets and to minimize the confounding influence of batch effects, the statistical associations between genes and clinical outcomes were assessed by z-scores. Combined z-scores represent the integrated gene expression data with clinical outcome from the public domain. Expression levels of MAFF and CPT2 across different breast cancer subtypes were retrieved from The Cancer Genome Atlas (TCGA) (https://www.cancer.gov/tcga) datasets and plotted. The correlation between MAFF and CPT2 was analyzed using METABRIC datasets based on mRNA expression z-scores normalized across all samples. Overall survival of basal-like breast cancer patients in relation to MAFF and CPT2 mRNA expression was analyzed using Kaplan-Meier plotter (KM plotter) [31, 32], which established survival data by combining multiple independent datasets with clinical data and gene expression measurements. TMA slide was purchased from Biomax (BR881). As all datasets and tissue samples used in this study were publicly available or commercially obtained and fully anonymized, this research was exempt from institutional ethics review.

### Statistical analysis

The data are presented as the mean, the standard deviation, and the individual observations of at least three replicates. The data were analyzed using GraphPad Prism software (GraphPad Prism 10, Dotmatics) as indicated in each figure. Briefly, for comparisons between two groups, we performed two-tailed Student’s *t*-tests. To compare multiple groups, we used One-way ANOVA for single-condition experiments and Two-way ANOVA for experiments involving multiple conditions. A *p* value of <0.05 was considered statistically significant.

## Results

### MAFF expression is associated with poor prognosis in breast cancer

**Fig. 1:**
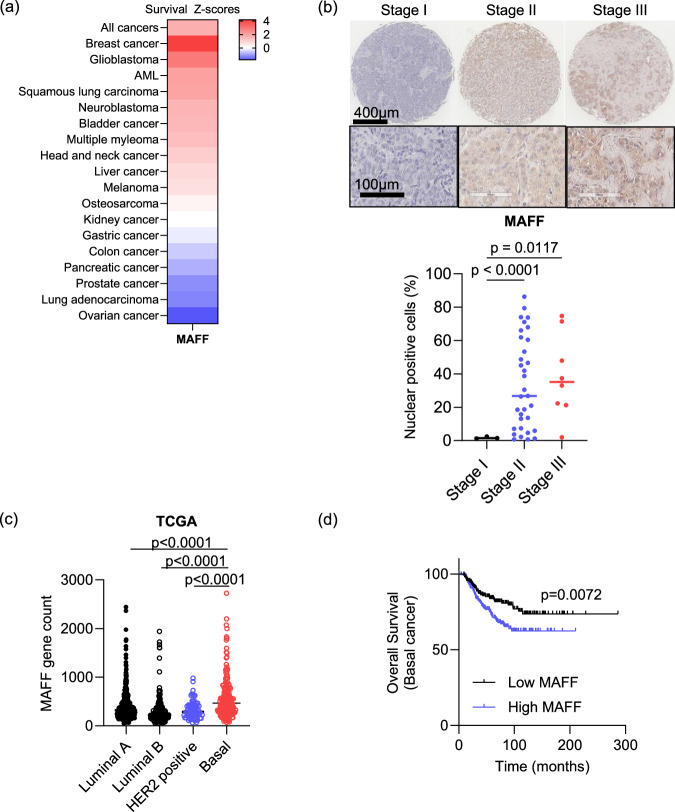
MAFF expression is associated with poor prognosis in breast cancer. **a** Meta-Z survival scores of MAFF across several cancer types generated using the PRECOG platform show a strong association with poor survival in breast cancer. **b** Analysis of nuclear MAFF expression in a breast cancer patient tissue microarray showed that MAFF expression correlates with advanced stage II and III disease (Top: Representative images at low magnification (scale bar = 400 μm), Bottom: Higher-magnification images (scale bar = 100 μm), Stage I: *n* = 3, Stage II: *n* = 33, Stage III: *n* = 8). **c** TCGA RNA-seq analysis of MAFF expression across breast cancer subtypes, showing higher expression in basal-like tumors (Luminal A: *n* = 499, Luminal B: *n* = 197, HER2 positive: *n* = 78, Basal: *n* = 171). **d** Kaplan–Meier survival analysis of basal-like breast cancer patients stratified into high vs low expression of MAFF using KM plotter demonstrated reduced overall survival with high MAFF expression (Low MAFF: *n* = 216, High MAFF: *n* = 215). Statistical tests were performed by One-way ANOVA with multiple comparison (**b**, **c**) and Log-rank test (**d**).

To strengthen our previous findings on the prognostic relevance of MAFF [12], we analyzed the association between *MAFF* expression and patient survival outcomes. A Meta-Z analysis of the PREdiction of Clinical Outcomes from Genomic Profiles (PRECOG) dataset [30] identified MAFF as a strong predictor of poor survival in multiple cancer types, with the most pronounced effect in breast cancer (Fig. 1a). Supporting this, breast cancer patient tissue microarray analysis revealed a marked increase in nuclear MAFF expression in Stage II and III tumors, indicating its link to disease progression (Fig. 1b). Subtype-specific The Cancer Genome Atlas (TCGA) data further showed that MAFF expression was selectively enriched in basal-like breast cancers compared to luminal A/B and HER2-positive tumors (Fig. 1c). Importantly, Kaplan–Meier survival analysis (KM plotter) [31, 32] demonstrated that high MAFF expression was significantly correlated with poor overall survival in basal-like breast cancer patients (Fig. 1d). Together, these findings establish MAFF not only as a marker of aggressive breast cancer but also as a potential driver of adverse clinical outcomes.

### MAFF expression increases susceptibility to ferroptosis

**Fig. 2:**
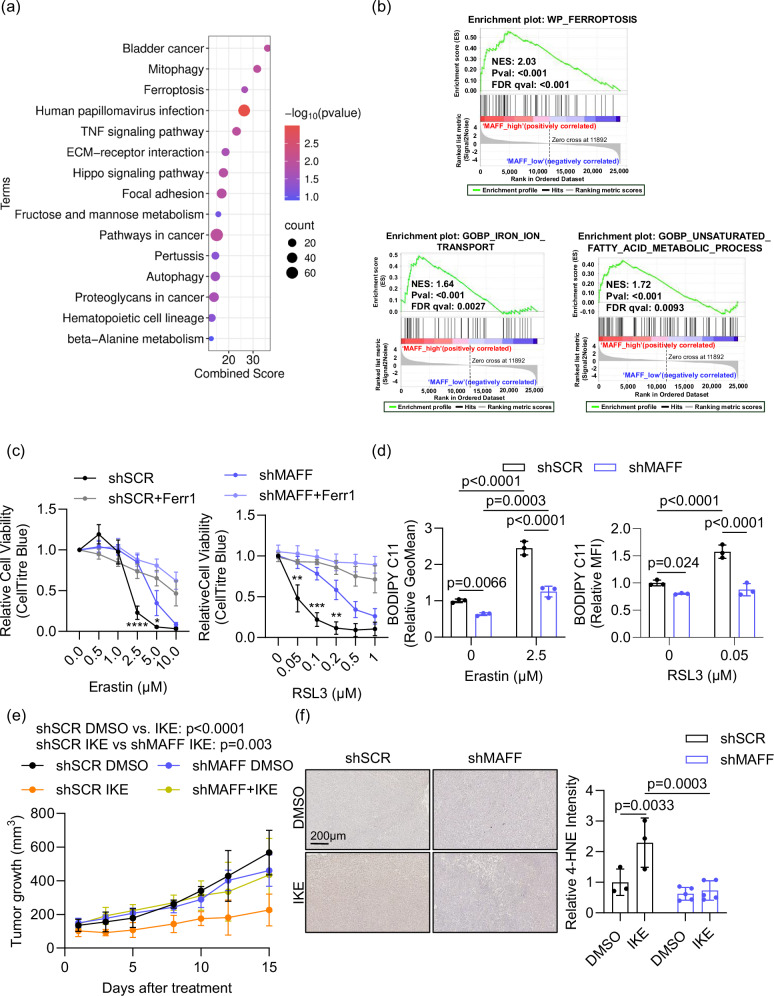
MAFF expression increases susceptibility to ferroptosis. **a** KEGG pathway analysis of direct target of MAFF from combined downregulated RNA- and ChIP-sequencing data revealed significant downregulation of genes in cancer-related and metabolic pathways, as well as the “Ferroptosis” pathway. **b** GSEA identified MAFF-dependent transcriptional programs linked to the “Ferroptosis,” “Unsaturated Fatty Acid Metabolism,” and “Iron Ion Transport”. **c** MAFF depletion (shMAFF) reduced erastin- and RSL3-induced ferroptotic death after 24 h incubation in MDA-MB-231 cells, as measured by CellTiter-Blue®, while Ferr-1 rescued viability, confirming ferroptosis-specific cytotoxicity. (*n* = 4, ****p < 0.0001, ***p = 0.0006, **p = 0.0036) **d** Flow cytometry analysis of BODIPY C11 revealed that erastin (2.5 μM)- or RSL3 (0.05 μM)-induced lipid peroxidation after 24 h incubation was diminished in the absence of MAFF (*n* = 3). **e** In orthotopic xenografts, IKE treatment suppressed control tumor growth but had minimal effect in MAFF-deficient tumors, indicating that MAFF is required for ferroptosis sensitivity in vivo (shSCR: *n* = 3, shMAFF: *n* = 5). **f** Lipid peroxidation, evaluated by 4-HNE staining, was significantly elevated by IKE treatment in MAFF-expressing tumors. Data are presented as means ± SD. Statistical tests were performed by One-way ANOVA (**c**) and Two-way ANOVA (**d**, **e**, **f**) with or without multiple comparisons.

To explore the pathways in which MAFF plays a critical role, we used our previously established MDA-MB-231 cells with MAFF knockdown (shMAFF) and its corresponding non-targeting control (shSCR, Fig. S1a) to perform RNA sequencing [12]. Direct transcriptional targets of MAFF showing significant expression changes (>1.3-fold) were identified by integrating RNA-sequencing data with previously generated MAFF ChIP-sequencing results [12]. We detected 1806 downregulated and 1866 upregulated genes as direct MAFF targets (Fig. S1b and Supplementary Table 1). KEGG pathway enrichment analysis using Enrichr [22–24] revealed that MAFF predominantly downregulated genes linked to cancer-related pathways, including “Bladder Cancer,” “TNF Signaling,” “Hippo Signaling,” “ECM–Receptor Interaction,“ and “Pathways in Cancer” (Fig. 2a). Metabolic pathways such as “Fructose and Mannose Metabolism” and “beta-Alanine Metabolism” were also affected. Interestingly, MAFF expression significantly influenced the “Ferroptosis” pathway, with the analysis showing that both ferroptosis inducers and repressors were downregulated by MAFF knockdown. (Figure S1c and Supplementary Table 2). In contrast, genes involved in the “Fatty acid elongation” pathway were upregulated following MAFF knockdown (Fig. S1d). Gene set enrichment analysis (GSEA) of all altered genes also highlighted enrichment of MAFF-regulated genes in the “Ferroptosis” pathway and related processes, including “Unsaturated Fatty Acid Metabolism and” “Iron Ion Transport,” (Fig. 2b).

To investigate whether MAFF could be a key regulator of ferroptosis, control and MAFF knockdown MDA-MB-231 cells were treated with the ferroptosis inducers erastin (SLC7A11 inhibitor) and 1S,3R-RSL-3 (RSL3, GPX4 inhibitor). In control cells (MDA shSCR), both erastin and RSL3 induced significant tumor cell death, which was reversed by the treatment of ferrostatin-1 (Ferr-1), a ferroptosis inhibitor [1, 4] (Fig. 2c). In contrast, MAFF knockdown (MDA shMAFF) reduced cell susceptibility to ferroptosis, resulting in decreased cell death. Consistently, lipid peroxidation measured by BODIPY C11 fluorescence was markedly increased by ferroptosis inducers in control cells, but this effect was abrogated in MAFF-deficient cells (Fig. 2d). Consistently, mitochondrial lipid peroxidation was also significantly reduced in MAFF knockdown cells compared with control cells, further supporting a role for MAFF in promoting ferroptosis-associated oxidative damage (Fig. S2a). To address potential off-target effects associated with single shRNA use, similar effects were confirmed using transient MAFF knockdown with an siRNA pool (Fig. S2b–d). Additionally, propidium iodide (PI) uptake assays confirmed that erastin induced ferroptotic cell death in control cells in a dose-dependent manner, which was significantly suppressed by Ferr-1 treatment, whereas MAFF knockdown markedly attenuated PI-positive cell death, excluding proliferation-related artifacts (Fig. S2e). Reduced sensitivity to ferroptosis upon MAFF depletion was also evident in two additional TNBC cell lines, BT549 and Hs578T, following erastin treatment (Fig. S2f–h). Conversely, MAFF overexpression in MDA-MB-231 cells (MAFF OE), which we previously generated [12], enhanced sensitivity to ferroptosis inducers, including erastin and RSL3, and further increased lipid peroxidation (Fig. S2i–k). These findings suggest that MAFF expression modulates ferroptotic sensitivity in TNBC cells.

To investigate the role of MAFF in the context of the tumor microenvironment, in vivo studies were conducted. MDA-MB-231 cells with or without MAFF expression were orthotopically injected into the mammary fat pads of athymic nude mice. Once tumors reached approximately 100 mm³, mice were treated with imidazole ketone erastin (IKE), a metabolically stable and more potent analogue of erastin, optimized for in vivo studies [33]. After 2-week IKE injection, a significant reduction in control tumor growth was observed (Fig. 2e). However, in the absence of MAFF, the growth of tumors remained unchanged, exhibiting consistent results compared to our in vitro studies. Immunohistochemical analysis of tumor tissues with 4-hydroxynonenal (4-HNE), a marker of lipid peroxidation, exhibited increased lipid peroxidation in control tumors following IKE treatment, whereas no significant changes were observed in tumors with MAFF knockdown (Figs. 2f and S2l).

### MAFF modulates ferroptotic sensitivity through regulation of intracellular iron levels

**Fig. 3:**
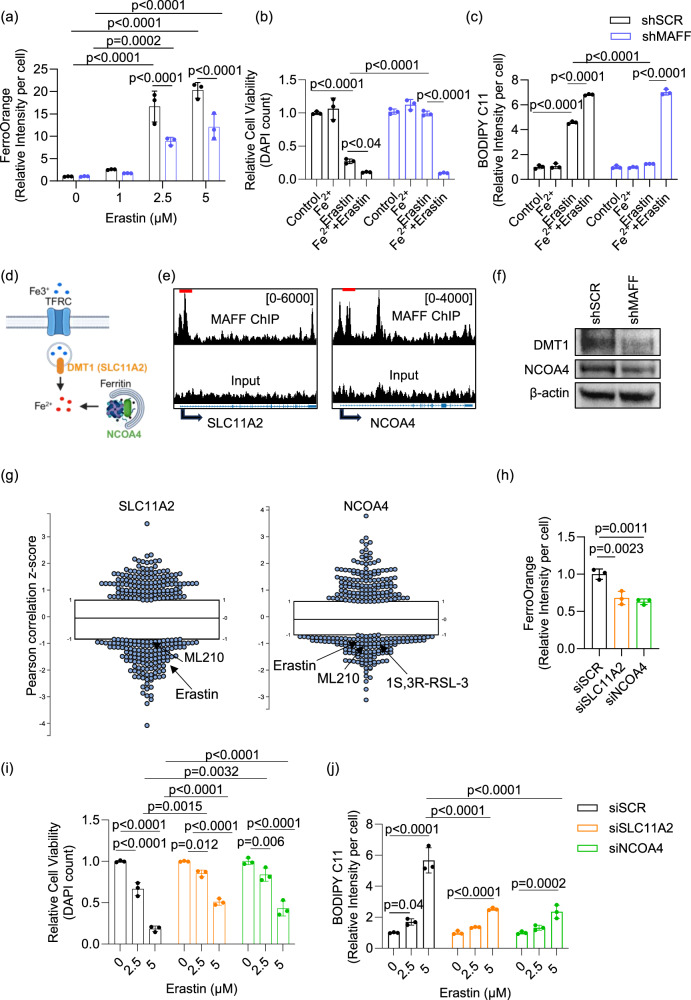
MAFF modulates ferroptotic sensitivity through regulation of intracellular iron levels. **a** Intracellular ferrous iron levels measured by FerroOrange increased with erastin treatment for 24 h in a dose-dependent manner in MDA-MB-231 cells. Compared to control cells, MAFF knockdown significantly reduced iron accumulation (*n* = 3). **b**, **c** Celigo analysis of DAPI-stained cells and C11-BODIPY lipid peroxidation revealed that treatment with 50 µM ammonium iron (II) sulfate combined with 2.5 µM erastin for 24 h promoted ferroptotic death in MDA-MB-231 cells, which was potentiated upon MAFF knockdown (*n* = 3). **d** Schematic representation of pathways influencing intracellular ferrous iron levels, including iron import via TFRC, reduction of ferric (Fe³⁺) to ferrous (Fe²⁺) iron by DMT1, and ferritinophagy-mediated iron release via NCOA4. **e** MAFF ChIP-sequencing profiles revealed strong binding at the promoter regions of *SLC11A2* and *NCOA4*. **f** Western blot analysis showed decreased DMT1 and NCOA4 protein expression upon MAFF knockdown. **g** Analysis of the CTRP demonstrated that expression of *SLC11A2* and *NCOA4* significantly correlated with sensitivity to ferroptosis inducers (erastin, M210, and 1S,3R-RSL-3). **h** Intracellular ferrous iron levels measured by FerroOrange decreased with knockdown of *SLC11A2* or *NCOA4* compared to control (siSCR). **i**, **j** Knockdown of *SLC11A2* or *NCOA4* reduced erastin-induced ferroptotic cell death (DAPI) and lipid peroxidation (BODIPY C11) after 24 h, as measured by Celigo (**i**, **j**). (*n* = 3). Data are presented as means ± SD. Statistical significance was determined by Two-way ANOVA (**a**–**c**, **i**–**j**) and One-way ANOVA (**h**) with multiple comparisons.

As an iron-dependent process, ferroptosis is profoundly affected by proteins that regulate iron transport, storage, and utilization [34], and, hence, we investigated whether MAFF modulates iron metabolism. In control cells, erastin treatment markedly increased intracellular ferrous iron (Fe²⁺) levels (Figs. 3a and S3a). While basal labile iron levels remained unchanged, MAFF knockdown using either shRNA or an siRNA pool significantly reduced the erastin-induced increase in ferrous iron. To determine whether reduced labile iron levels in MAFF knockdown cells contributed to the diminished ferroptosis response, exogenous iron in the form of iron (II) sulfate, was added to both control and MAFF knockdown cells. Iron (II) sulfate alone did not affect cell viability or lipid peroxidation in the presence of MAFF, but it significantly increased ferroptosis sensitivity when MAFF was inhibited, indicating that MAFF modulates susceptibility to ferroptosis by regulating iron availability (Fig. 3b, c).

To identify the target genes through which MAFF regulates ferroptosis, we examined ferroptosis-related genes within the KEGG pathways significantly influenced by MAFF expression. This analysis revealed a mix of ferroptosis-related genes, including both inducers and repressors, were downregulated by MAFF knockdown (Fig. S1c and Supplementary Table 2). We focused on genes whose expression patterns correlated with MAFF in response to ferroptosis. To identify direct targets of MAFF, we further refined the gene list by selecting those with prominent binding peaks enriched around transcriptional start sites (TSS) in our previous ChIP sequencing data [12]. Interestingly, two genes (*SLC11A2, NCOA4*) involved in iron homeostasis were significantly regulated by MAFF (Fig. 3d–f). Knocking down *MAFF* led to decreased expression of *SLC11A2* and *NCOA4*, indicating that MAFF is a transcriptional activator of these genes (Figs. 3f and S3b). Both *SLC11A2*, which encodes the divalent metal transporter 1 (DMT1), and NCOA4 contribute to the release of ferrous iron into the cytosolic labile iron pool, facilitating the Fenton reaction, either from the endosome or autophagosome [35–37]. Data from the Cancer Therapeutic Response Portal (CTRP) further showed that their expression was significantly correlated with drug sensitivity to ferroptosis inducers (Erastin, M210, and 1S,3R-RSL-3, Fig. 3g). The role of both *SLC11A2* and *NCOA4* in ferroptosis was further confirmed by knocking down these genes using siRNAs (Fig. S3c). While silencing these genes significantly reduced ferrous iron levels, erastin-induced cell death and lipid peroxidation were also attenuated. (Fig. 3h–j). Therefore, our data indicate that MAFF enhances ferroptotic sensitivity by increasing the labile iron pool through regulation of DMT1 (*SLC11A2*) and NCOA4.

### MAFF enhances ferroptotic sensitivity through lipid metabolic reprogramming

**Fig. 4:**
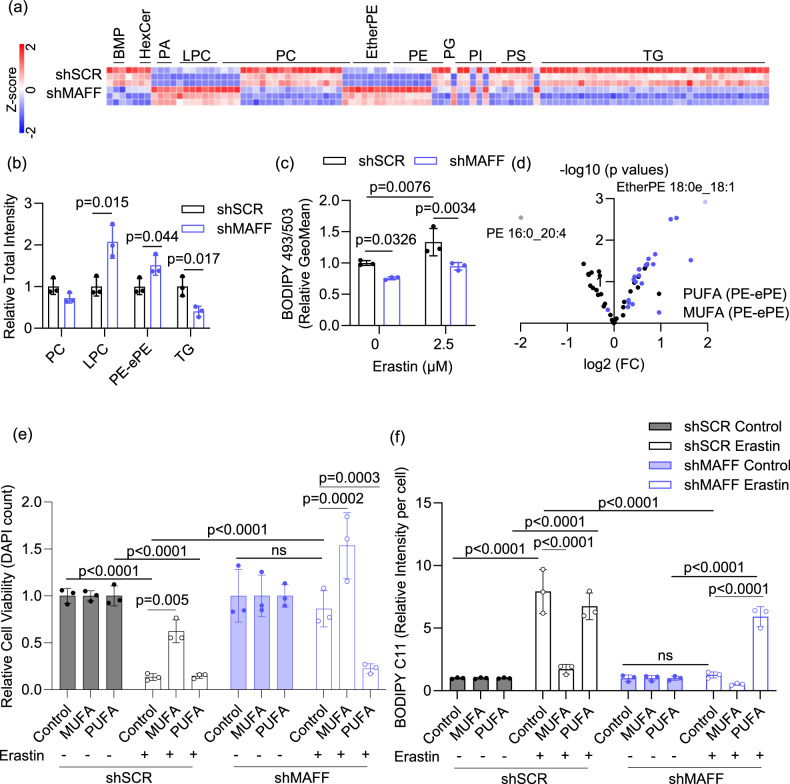
MAFF enhances ferroptotic sensitivity through lipid metabolic reprogramming. **a** Lipidomic profiling of MDA-MB-231 cells following MAFF depletion, shown as a heatmap of relative changes in major lipid class intensities. **b** The total intensities, normalized to the control group, revealed a trend toward decreased PC, with significant increases in LPC and PE/etherPE (ePE), whereas TG was significantly reduced (*n* = 3). **c** Lipid droplet content, measured by BODIPY 493/503 staining and flow cytometry, was significantly reduced upon MAFF knockdown both at baseline and following 24 h erastin treatment. (*n* = 3) **d** Analysis of PE and etherPE species showed a shift from PUFA toward MUFA in the absence of MAFF. **e**, **f** Supplementation with MUFA (oleic acid 200 µM) protected control cells from erastin-induced ferroptosis, as evidenced by increased survival (**d**, DAPI) and reduced lipid peroxidation (**e**, BODIPY C11) after 24 h of treatment measured by celigo, whereas PUFA treatment (linoleic acid 400 µM) enhanced ferroptosis in MAFF-deficient cells (*n* = 3). Data are presented as means ± SD. Statistical significance was determined by Multiple upaired *t*-tests (**b**) and Two-way ANOVA with multiple comparisons (**c**, **e**, **f**).

As ferroptosis is driven by the peroxidation of PUFAs, pathways involved in lipid metabolism and fatty acid biogenesis play a critical role in regulating this cell death process [38–41]. Building on our findings from the KEGG and GSEA analysis (Figs. 2a, b and S1c and Supplementary Table 2), we examined whether MAFF expression influenced fatty acid content in cells. Untargeted lipidomic profiling was performed using LC–QTOF–MS. Heatmap results revealed broad alterations across multiple lipid classes following MAFF knockdown (Fig. 4a). Although the total intensity of phosphatidylcholine (PC) showed a decreasing trend, the total intensities of lysophosphatidylcholine (LPC) and combined phosphatidylethanolamines (PE and etherPE) were significantly increased, suggesting enhanced phospholipid turnover or membrane remodeling (Fig. 4b). Notably, triglycerides (TG) exhibited the most pronounced reduction. As TGs are essential for lipid droplet (LD) formation, we observed that basal levels of LD were lower on MAFF knockdown cells generated using either shRNA or an siRNA pool compared to control (Figs. 4c and S4a). Moreover, erastin treatment induced LD accumulation in control cells, but this effect was abolished in MAFF-depleted cells, indicating a sustained suppression of LD formation. Perilipin 3 (PLIN3) is a member of the PAT family of LD-associated proteins that plays a key role in LD formation and stability by binding to nascent LDs and influencing neutral lipid storage dynamics [42]. ChIP-sequencing analysis revealed MAFF binding at the PLIN3 TSS, and protein levels of PLIN3 were markedly reduced upon MAFF knockdown (Figs. S4b and S4c). These results indicate that MAFF directly regulates PLIN3 expression, thereby linking MAFF-dependent transcriptional control to LD homeostasis.

Interestingly, loss of MAFF also altered the lipid landscape, shifting it from PUFAs toward monounsaturated fatty acids (MUFAs) (Fig. 4d). This shift was most apparent in the increased abundance of MUFA-containing etherPE species, accompanied by a reduction in PUFA-containing PE16:0_20:4, the most susceptible lipid to lipid peroxidation [43]. As previously reported, the balance between PUFAs and MUFAs is a critical determinant of ferroptosis sensitivity [3, 20, 44]. To investigate whether MAFF-dependent regulation of PUFA-MUFA balance contributes to ferroptosis, we treated MDA-MB-231 cells with either oleic acid (OA, MUFA) or linoleic acid (LA, PUFA) in the presence and the absence of MAFF. In shSCR cells, the addition of OA reduced erastin-induced cell death and lipid peroxidation, whereas shMAFF cells were largely resistant to erastin-induced ferroptosis regardless of OA treatment (Fig. 4e, f). Conversely, LA treatment significantly increased cell death and lipid peroxidation in shMAFF cells but did not cause further changes in shSCR cells. These findings indicate that MAFF sensitizes tumors to ferroptosis by regulating PUFAs.

### MAFF represses genes in fatty acid oxidation and synthesis

**Fig. 5:**
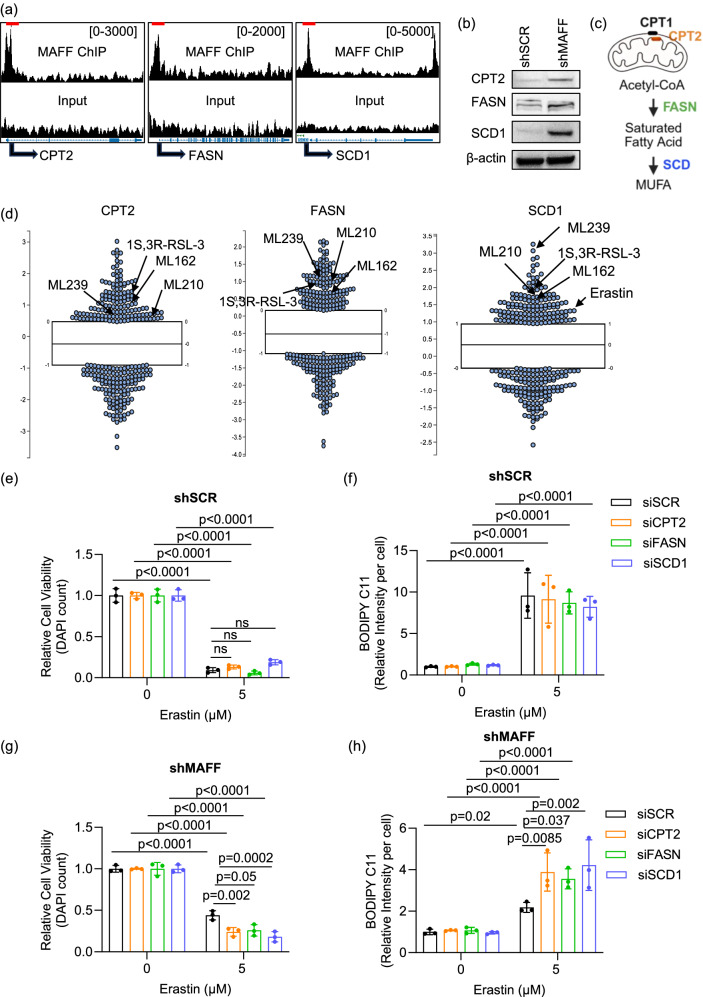
MAFF represses genes in fatty acid oxidation and synthesis. **a** MAFF ChIP-sequencing data presented strong binding at the promoter regions of *CPT2, FASN*, and *SCD1*. **b** MAFF knockdown increased protein expression of CPT2, FASN, and SCD1 measured by western blot. **c** Schematic representation of pathways regulated by CPT2, FASN, and SCD1 **d** The CTRP analysis showed that expression of *CPT2*, *FASN*, and *SCD1* inversely correlated with sensitivity to ferroptosis inducers (erastin, M210, ML162, ML239, and 1S,3R-RSL-3). **e**, **f** In control MDA-MB-231 cells, knocking down *CPT2*, *FASN*, and *SCD1* using siRNApool showed similar levels of ferroptosis induction measured by cell survival and lipid peroxidation after 24 h of erastin treatment using Celigo (*n* = 3). **g**, **h** In the absence of MAFF, knocking down *CPT2*, *FASN*, and *SCD1* sensitized tumor cells to erastin-induced ferroptosis measured by cell survival and lipid peroxidation after 24 h using Celigo (*n* = 3). Data are presented as means ± SD. Statistical significance was determined by Two-way ANOVA with multiple comparisons (**e**–**h**).

To further identify MAFF target genes involved in lipid and fatty acid metabolism, we focused on genes within fatty acid metabolic pathways that exhibited distinct MAFF binding peaks near their TSS. We found MAFF binding in the TSS of *CPT2*, *FASN*, and *SCD1* genes (Fig. 5a). Notably, these genes were transcriptionally repressed by MAFF (Figs. 5b and S5a). Carnitine palmitoyltransferase 2 (CPT2) is a key mitochondrial enzyme in long-chain fatty acid β-oxidation that converts imported acylcarnitines back into acyl-coenzyme A (acyl-CoA) within the mitochondrial matrix, allowing their subsequent oxidation to acetyl-CoA (Fig. 5c) [45]. From acetyl-CoA, fatty acid synthase (FASN) generates long-chain saturated fatty acids, which are then converted to MUFAs by stearoyl-CoA desaturase 1 (SCD1), a rate-limiting enzyme in MUFA biosynthesis [46]. CTRP analysis showed the inverse correlation between these genes and sensitivity to several ferroptosis inducers (Fig. 5d). To test whether MAFF-dependent repression of *CPT2*, *FASN*, and *SCD1* contributes to ferroptosis susceptibility, we knocked down these genes in both shSCR and shMAFF cells (Fig. S5b). In control (shSCR) cells, which were already sensitive to ferroptosis, gene knockdown did not further increase erastin-induced cell death or lipid peroxidation (Fig. 5e, f). However, in shMAFF cells, knockdown of *CPT2*, *FASN*, or *SCD1* markedly enhanced ferroptosis, indicating that MAFF protects against ferroptosis by regulating this metabolic axis (Fig. 5g, h).

### CPT2 is a negative regulator of tumor invasion and progression

**Fig. 6:**
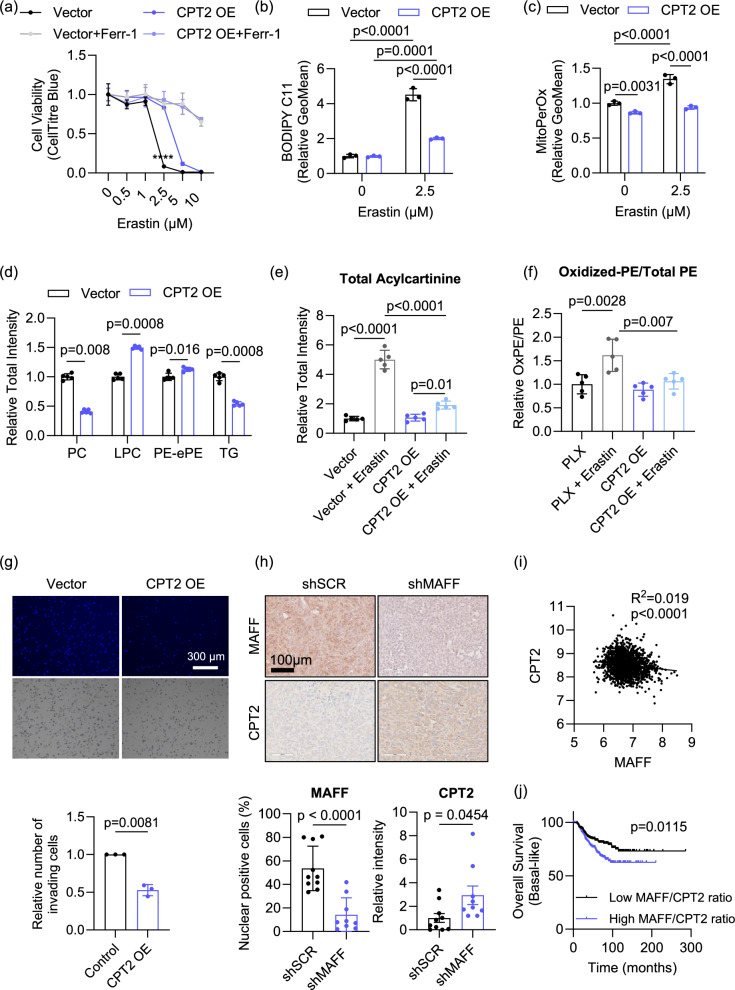
CPT2 is a negative regulator of tumor invasion and progression. Overexpression of CPT2 in MDA-MB-231 cells significantly enhanced erastin-induced cell death after 24 h, as measured by the CellTiter-Blue®, assay (**a**, *n* = 3), and increased lipid peroxidation and mitochondrial lipid peroxidation levels, determined by BODIPY-C11 (**b**, *n* = 3) and MitoPerOx (**c**, *n* = 3) fluorescence using flow cytometry. **d** Total intensities, normalized to the control group, indicated that CPT2 overexpression significantly decreased PC and TG, while increasing LPC and PE-etherPE (ePE) (*n* = 5). **e** Mean acylcarnitine intensities indicated that CPT2 overexpression did not alter basal levels of acylcarnitine but attenuated the increase of acylcarnitine upon erastin treatment (*n* = 5). **f** Ratio of oxidized-PE/total PE indicated that only erastin-treated vector control cells after 24 h had high oxidation of PE (*n* = 5). **g** Invasion assays using Matrigel-coated invasion chambers demonstrated that CPT2 overexpression markedly decreased tumor cell invasion. Cells were visualized by DAPI staining (upper panels) or with a three-step staining kit (bottom panels, *n* = 3). **h** Tumor tissues derived from MDA-MB-231 mouse xenografts exhibited a significant increase in CPT2 expression upon MAFF knockdown (shSCR; *n* = 10, shMAFF: *n* = 9). **i** METABRIC dataset analysis of mRNA z-scores showed a significant inverse correlation between MAFF and CPT2 expression in breast cancer patients (*n* = 1866). **j** A high MAFF-to-CPT2 expression ratio was associated with poorer overall survival among basal-like breast cancer patients, as shown by KM plotter analysis (Low MAFF/CPT2 ratio: *n* = 216, High MAFF/CPT2 ratio: n = 215). Data are presented as means ± SD. Statistical significance was determined by Two-way ANOVA with multiple comparisons (**a**–**c**, **e**, **f**), Multiple unpaired *t*-tests (**d**), unpaired *t*-test with Welch’s correction (**g**, **h**), simple linear regression (**i**), and log-rank (**j**).

Our data demonstrate that MAFF increases tumor susceptibility to ferroptosis through direct repression of CPT2 expression. Although a direct role of CPT2 in ferroptosis has not been clearly established, our findings provide functional evidence that CPT2 modulates ferroptosis sensitivity. Consistently, direct inhibition of CPT2 phenocopied MAFF overexpression, leading to a significant increase in ferroptosis sensitivity. (Fig. 5g, h). In contrast to CPT2 knockdown, CPT2 overexpression (Fig. S6a) suppressed ferroptotic responses in breast cancer cells, as evidenced by reduced cell death (Fig. 6a), lipid peroxidation (Fig. 6b), and mitochondrial lipid peroxidation (Fig. 6c). To determine whether CPT2 overexpression contributes to lipid reprogramming associated with reduced ferroptosis sensitivity, we next performed a lipidomic analysis. In CPT2-overexpressing (CPT2 OE) cells, the total intensity of PC was significantly decreased (Fig. 6d). Consistent with the shMAFF, LPC and PE levels were significantly increased, whereas TG levels were decreased. Lipidomic analysis showed that CPT2 overexpression alone did not significantly affect acylcarnitine levels under basal conditions (Fig. 6e). However, erastin treatment markedly increased acylcarnitine levels in vector control cells, whereas this increase was attenuated in CPT2-overexpressing cells. These results suggest that CPT2 modulates acylcarnitine accumulation in response to ferroptotic stress. In contrast to shMAFF, CPT2 overexpression did not induce a shift toward MUFA-enriched PE/etherPE species. Instead, both MUFA- and PUFA-containing PE and etherPE species were concomitantly increased (Fig. S6b). Despite this overall increase, the oxidized-PE/total PE ratio was markedly elevated only in erastin-treated vector control cells (Fig. 6f). Notably, this increase was largely suppressed in CPT2-overexpressing cells, consistent with reduced ferroptosis-associated lipid peroxidation.

Additionally, we observed that the overexpression of CPT2 also led to a marked decrease in tumor cell invasion, linking this pathway to cancer progression (Fig. 6g). To assess whether our in vitro findings translate to a more complex in vivo system, we examined tumor tissues from prior mouse studies demonstrating that MAFF knockdown suppresses lung metastasis [12]. CPT2 expression was significantly increased in primary tumors derived from MAFF-knockdown cells compared with controls (Fig. 6h). Tying these observations together, MAFF expression was inversely correlated with CPT2 expression in breast cancer patients (Fig. 6i).

Complementing our experimental data, a Meta-Z analysis using the PRECOG database revealed the clinical significance of CPT2 (Fig. S6c), highlighting its role as a tumor suppressor, which has been previously noted in liver, ovarian, and renal cancers [47–49]. In contrast to MAFF, higher CPT2 expression was associated with a better prognosis across numerous cancer types, including breast cancer. Within breast cancer subtypes, CPT2 expression was notably lower in aggressive basal-like tumors compared to luminal A/B or HER2-positive types (Fig. S6d). Consequently, a high MAFF/CPT2 ratio was significantly correlated with poor overall patient survival, indicating that MAFF-mediated CPT2 repression regulates tumor progression (Fig. 6j). These findings align with our central hypothesis that MAFF-mediated repression of CPT2 is a crucial mechanism that simultaneously modulates ferroptosis and enhances the invasive potential of tumor cells.

## Discussion

We previously identified MAFF as a key regulator of tumor invasion and metastasis in breast and ovarian cancers [12]. In this study, we further uncover that while MAFF promotes a poor prognosis of basal-like breast cancer, it simultaneously sensitizes these aggressive cells to ferroptosis by reprogramming their iron and lipid metabolism through transcriptional regulation of key metabolic genes. The enhanced sensitivity of invasive and drug-resistant tumor types to ferroptosis has attracted significant interest as a potential therapeutic strategy for treating aggressive cancers [4, 6–11, 50]. Interestingly, this ferroptotic vulnerability often arises from the same pathways that drive malignancy. For instance, transcription factors such as ZEB1 and HIF2, which are vital for metastatic survival and hypoxia adaptation, can also render cells more susceptible to ferroptosis [6, 50]. Similarly, the enzyme acyl-CoA synthetase long-chain family member 4 (ACSL4) enhances ferroptosis by promoting the incorporation of PUFAs into cellular membranes [51]. This PUFA enrichment not only primes cells for lipid peroxidation and ferroptotic cell death but also increases membrane fluidity, thereby enhancing the metastatic potential of tumor cells [10, 11]. Consistent with these paradigms, our findings demonstrate that the transcription factor MAFF acts as a transcriptional node linking pro-metastatic metabolic programs to increased ferroptosis sensitivity through coordinated regulation of iron and lipid metabolism.

Our discovery that MAFF is a regulator of ferroptosis is supported by emerging evidence from lung cancer, where MAFF was shown to regulate *SCL7A11* expression and influence ferroptotic sensitivity [52]. However, our findings further extend this observation substantially by revealing that MAFF functions as a central coordinator of ferroptosis by regulating iron and fatty acid availability [53–55]. A key finding of our study is the direct transcriptional control by MAFF over the labile iron pool. We show that MAFF transcriptionally activates essential genes for iron homeostasis, such as *SLC11A2* (DMT1) and *NCOA4*. By upregulating *SLC11A2*, an endosomal iron transporter [36] and *NCOA4*, the cargo receptor for ferritinophagy [37], MAFF supplies iron to initiate ferroptosis. In parallel, our work reveals that MAFF remodels the cellular lipid landscape by transcriptionally repressing key enzymes in fatty acid metabolism, including *CPT2*, *FASN*, and *SCD1*. The downregulation of *FASN* and *SCD1* is particularly important, as this combined suppression limits the synthesis of protective MUFAs, thereby shifting the balance towards an accumulation of highly peroxidizable PUFAs [46]. Additionally, our findings indicate that MAFF-mediated changes in TG levels influence LD formation. The role of LDs in ferroptosis is complex [20, 44, 50, 53, 56–58]. Indeed, studies in clear-cell carcinoma have shown that simple LD accumulation is not sufficient to drive ferroptosis susceptibility [50]. Rather, it is the enrichment of these LDs with PUFAs that makes them a key contributor, acting as a reservoir of lipids vulnerable to peroxidation. Consistent with this model, we observed a significant, MAFF-dependent accumulation of LDs following treatment with erastin. However, whether these LD changes directly contribute to ferroptosis or primarily reflect alterations in TG metabolism remains unclear and warrants further investigation in future studies.

Fatty acid oxidation (FAO), a core mitochondrial pathway for energy production, is initiated by the coordinated actions of carnitine palmitoyltransferases CPT1 and CPT2 [59]. The role of FAO in ferroptosis is increasingly recognized as context dependent. For instance, CPT1-mediated FAO can either sustain redox homeostasis and suppress lipid peroxidation by replenishing NADPH and glutathione pools [60] or, under conditions of impaired mitochondrial respiration, promote excessive lipid oxidation and sensitize cells to ferroptosis [61]. In contrast, the function of CPT2 in ferroptosis remains largely unexplored. Our identification of CPT2 among MAFF-repressed targets is particularly compelling, as it directly links the pro-ferroptotic function of MAFF to suppression of a metabolic enzyme with established tumor-suppressive properties. CPT2, a key enzyme in mitochondrial fatty acid β-oxidation, has been recognized for its tumor-suppressive roles, as its overexpression reduces invasion and metastasis in ovarian, renal, and liver cancers [47–49]. Consistent with these reports, our analysis further demonstrates that higher CPT2 expression correlates with improved clinical outcomes in basal-like breast cancer, in direct opposition to the prognostic signature of MAFF. Functionally, we provide evidence that CPT2 protects cells from ferroptosis, as its knockdown re-sensitized MAFF-deficient cells to ferroptosis induction, whereas CPT2 overexpression was associated with reduced sensitivity to ferroptotic cell death. Notably, we show for the first time that CPT2 overexpression is accompanied by a distinct remodeling of the lipid landscape, including alterations in the mean class intensities of PC, PE, and TG. Importantly, while CPT2 overexpression did not significantly alter the PUFA/MUFA ratio within PE species, it markedly reduced the oxidized-PE to PE ratio under ferroptotic stress, indicating suppression of lipid peroxidation. These findings suggest a CPT2-specific function in regulating ferroptosis-associated lipid peroxidation, independent of alterations in PUFA/MUFA composition. Interestingly, CPT2 overexpression suppressed tumor invasion, highlighting a complex and inverse relationship between ferroptosis sensitivity and tumor progression.

The dual role of MAFF as both a pro-metastatic factor and a ferroptosis sensitizer reveals a compelling “double-edged sword” paradigm in aggressive breast cancer. MAFF-driven metabolic reprogramming, while promoting invasion and adaptation, simultaneously primes tumors for ferroptotic vulnerability, creating a metabolic state that can be therapeutically exploited. Our findings indicate that high MAFF expression may serve as a predictive biomarker to identify basal-like breast cancer patients most likely to benefit from ferroptosis-inducing therapies. Targeting tumors already metabolically predisposed to ferroptosis could represent an effective strategy against this otherwise poorly responsive cancer subtype. In conclusion, our study highlights MAFF as a significant regulator of ferroptosis in breast cancer, mediated by transcriptional programs governing iron homeostasis and lipid composition. This work significantly advances our understanding of the metabolic underpinnings of aggressive breast cancer and provides a compelling rationale for a biomarker-driven therapeutic approach using ferroptosis inducers.

## Supplementary information

Supplementary Figure Legends

Supplementary Figures

Original Data

Supplementary Table 1

Supplementary Table 2

Supplementary Table 3

Supplementary Table 4

## Data Availability

Previously deposited ChIP-sequencing data are available under accession number GSE144964 at Gene Expression Omnibus (GEO). RNA-sequencing data for MDA-MB-231 shSCR and shMAFF are available under GSE275437 and GSE310349.
