# Supplementary material for: MAFF regulates ferroptotic sensitivity through iron homeostasis and fatty acid synthesis
Source: Cell Death Dis. 2026 May 28;17(1):656. doi: 10.1038/s41419-026-08885-w (PMC13402699; doi:10.1038/s41419-026-08885-w)
Supplement: Supplementary file 1 — Supplementary Figure Legends [file 41419_2026_8885_MOESM1_ESM.docx]

**Supplementary Table 1.** The list of MAFF target genes that were downregulated or upregulated upon MAFF knockdown overlapped between RNA- and ChIP-sequencing analyses.

**Supplementary Table 2.** KEGG pathway analysis of genes upregulated or downregulated by MAFF knockdown.

**Supplementary Table 3.** List of lipid classes identified by untargeted lipidomic analysis comparing shMAFF cells with non-targeted control cells (shSCR).

**Supplementary Table 4**. Median intensities of lipid classes identified by lipidomic analysis in control (Vector) and CPT2-overexpressing cells.

**Supplementary Figure Legends**

**Supplementary Figure 1**

(a) Western blot analysis confirming the downregulation of MAFF in the MAFF knockdown cell line compared to the control cell line. (b) Overlap of significantly downregulated (RNA-seq DOWN) or upregulated (RNA-seq UP) genes (≥1.3-fold change) with ChIP-sequencing MAFF binding genes. (c) Expression fold changes of KEGG *Ferroptosis* pathway genes in MAFF knockdown (shMAFF) cells relative to control (shSCR) cells. (d) KEGG pathway analysis of direct target of MAFF from combined upregulated RNA- and ChIP-sequencing data revealed that genes in cancer-related and metabolic pathways such as 'Fatty acid elongation' were significantly upregulated.

**Supplementary Figure 2**

(a) Mitochondrial lipid peroxidation, measured using MitoPerOx, was significantly increased following treatment with 2.5 µM erastin for 24 hours in control MDA-MB-231 cells (shSCR), whereas this increase was not observed in MAFF-deficient cells (shMAFF) (n = 3). (b-c) Celigo analysis of DAPI-stained cells and C11-BODIPY lipid peroxidation revealed that silencing MAFF (siMAFF) reduced erastin (2.5 µM) induced cell death (b) and lipid peroxidation induction (c) after 24 hours. (d) Western blot analysis confirming the downregulation of MAFF in the MAFF silenced cell line compared to the control cell line. (e) Erastin treatment for 24 hours increased PI uptake in control MDA-MB-231 (shSCR) cells in a dose-dependent manner, and this effect was significantly suppressed by co-treatment with Ferr-1. MAFF knockdown markedly reduced PI-positive cell death, (f-g) MAFF depletion (shMAFF) reduced erastin-induced ferroptotic death in BT549 (f) and Hs578T (g) cells 24 hours after treatment, as measured by CellTiter-Blue, while Ferr-1 rescued viability, confirming ferroptosis-specific cytotoxicity (n = 3, **** p<0.0001, *** p<0.001). (h) Western blot analysis confirming the downregulation of MAFF in the MAFF knockdown BT549 and Hs578T cell lines compared to the control cell line. (i-j) CellTiter-Blue viability (i) and Celigo analysis of C11-BODIPY lipid peroxidation (j) revealed that MAFF overexpression (MAFF OE) increased erastin- and RSL3-induced ferroptotic death in MDA-MB-231 cells after 24 hours of treatment, while Ferr-1 rescued viability, and MAFF OE also showed increased erastin- and RLS3-induced lipid peroxidation confirming ferroptosis-specific cytotoxicity (n = 3, **** p<0.0001, *** p<0.001). (k) Western blot analysis confirming the overexpression of MAFF tagged with V5 in the MAFF OE cell line. (l) IHC analysis of MAFF nuclear staining in shSCR and shMAFF tumor tissues confirmed effective MAFF knockdown in shMAFF tumors (shSCR: n = 3, shMAFF: n = 5). Data are presented as means ± SD. Statistical significance was determined by Two-way ANOVA with multiple comparison (a-c, e-g, i-j) and unpaired *t*-test with Welch’s correction (l).

**Supplementary Figure 3**

(a) FerroOrange-based measurement of intracellular iron levels showing a significant increase upon 24 hours treatment with 2.5 µM erastin, with no significant change following MAFF siRNA knockdown (n = 3). (b) Relative mRNA expression of *SLC11A2* and *NCOA4* in control vs MAFF knockdown cells, showing that both genes are downregulated upon MAFF knockdown. (c) Western blot analysis confirmed the silencing of DMT1 and NCOA4 in the control and MAFF knockdown cell lines (n = 3). Data are presented as means ± SD. Statistical significance was determined by Two-way ANOVA with multiple comparisons.

**Supplementary Figure 4**

(a) In control MDA-MB-231 cells (siSCR), treatment with 2.5 µM erastin for 24 hours significantly increased lipid droplet accumulation, as assessed by BODIPY 493/503 staining and flow cytometry. In contrast, MAFF knockdown using an siRNA pool reduced both basal lipid droplet levels and erastin-induced lipid droplet accumulation (n = 3). Data are presented as means ± SD. Statistical significance was determined by Two-way ANOVA with multiple comparisons. (b) MAFF ChIP-seq data presented strong binding at the promoter region of *PLIN3*. (c) Western blot analysis showed decreased PLIN3 protein expression upon MAFF knockdown.

**Supplementary Figure 5**

(a) Relative mRNA expression of *CPT2*, *SCD1* and *FASN* in control vs MAFF knockdown cells, showing that both genes are upregulated upon MAFF knockdown (n = 3). Data are presented as means ± SD. Statistical significance was determined by multiple unpaired t-tests. (b) Western blot analysis confirmed the silencing of CPT2, FASN and SCD1 in the control (shSCR) and MAFF knockdown (shMAFF) cell lines.

**Supplementary Figure 6**

(a) Western blot analysis confirmed the overexpression of CPT2 in the CPT2 OE cell line. (b) Lipidomic analysis revealed overall increases in both PUFA- and MUFA-containing PE and etherPE (ePE) species (n = 5). (c) PRECOG analysis revealed that Meta-Z survival scores for CPT2 across multiple cancer types, including breast cancer, were strongly associated with improved patient survival. (d) TCGA RNA-seq data showed lower CPT2 expression in basal-like breast tumors compared to other subtypes (Luminal A: n = 499, Luminal B: n = 197, HER2-positive: n = 78, Basal: n = 171).
