# Supplementary figures and images for: MAFF regulates ferroptotic sensitivity through iron homeostasis and fatty acid synthesis
Source: Cell Death Dis. 2026 May 28;17(1):656. doi: 10.1038/s41419-026-08885-w (PMC13402699; doi:10.1038/s41419-026-08885-w)

Fig. S1.

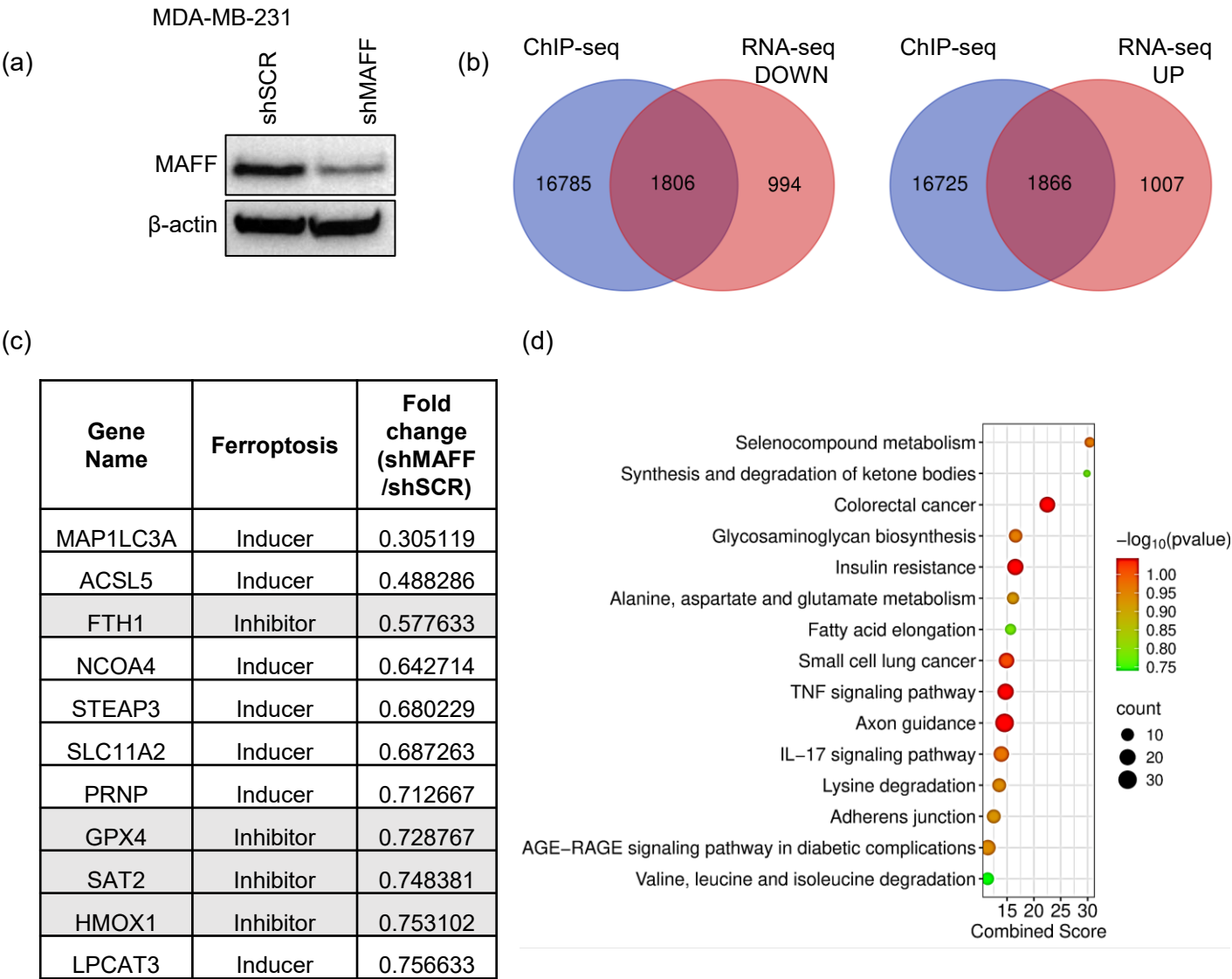

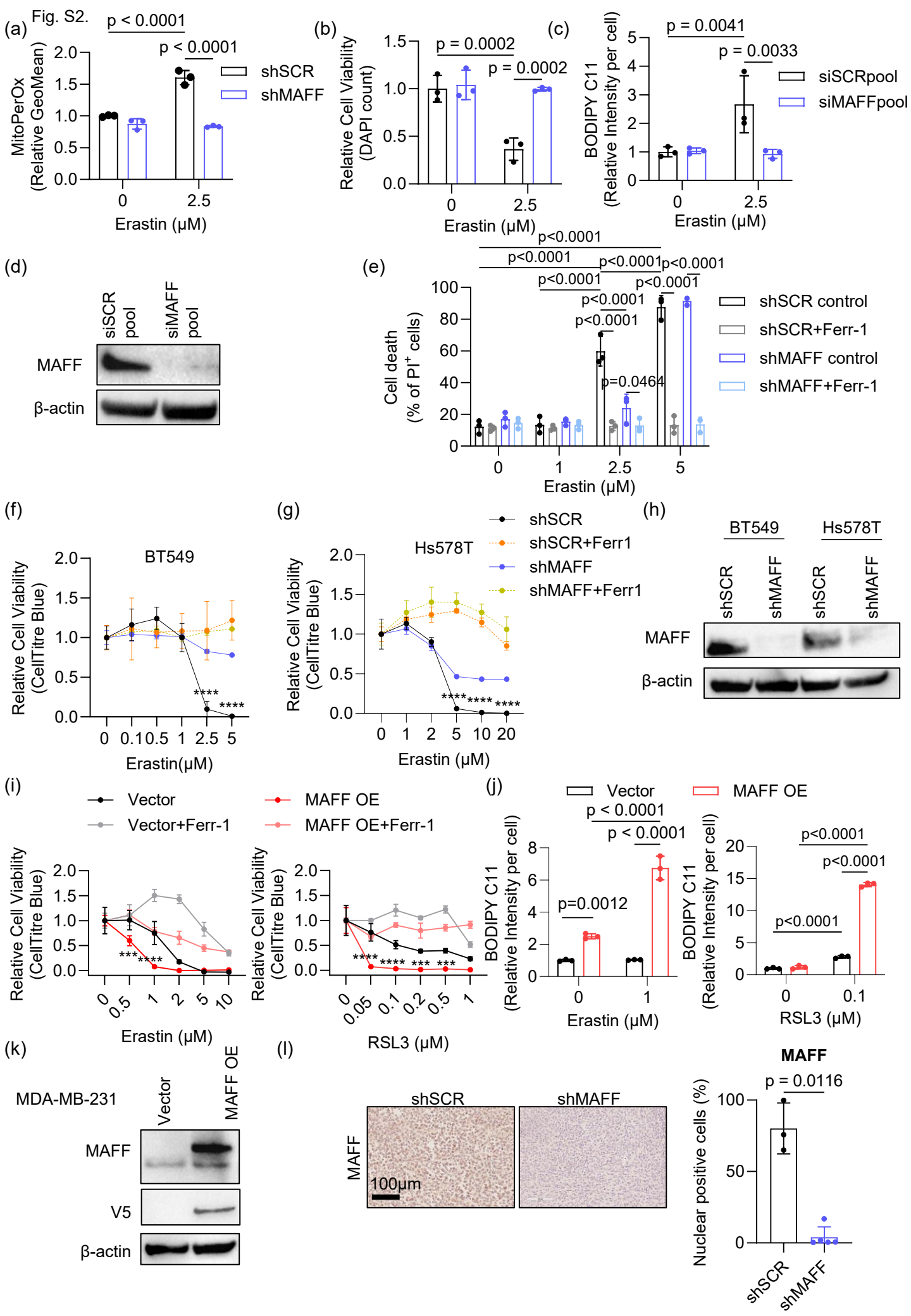

Fig. S3.

(a)

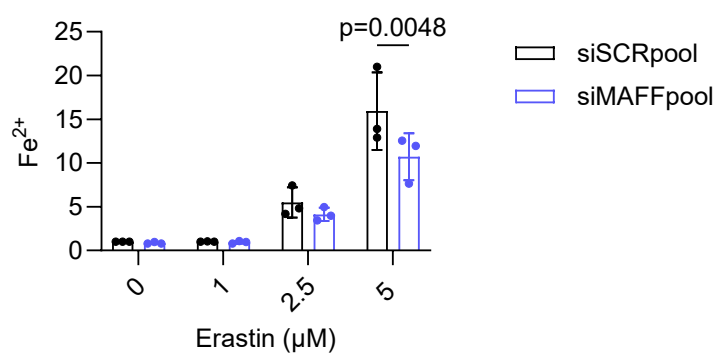

(b)

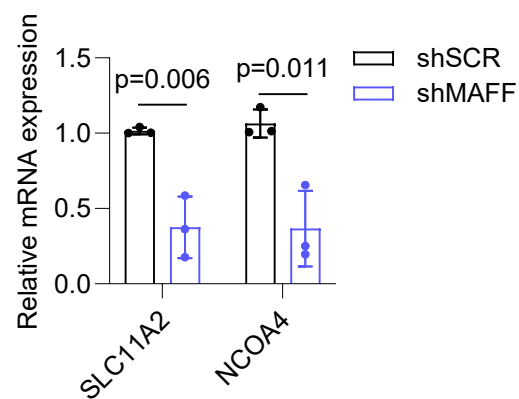

(c)

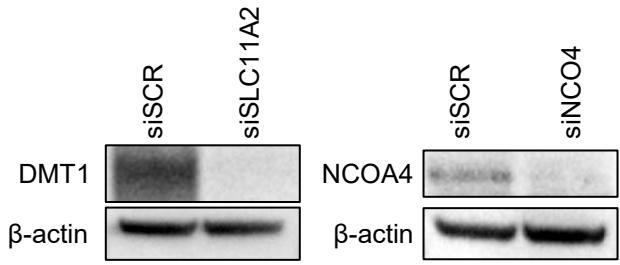

Fig. S4.

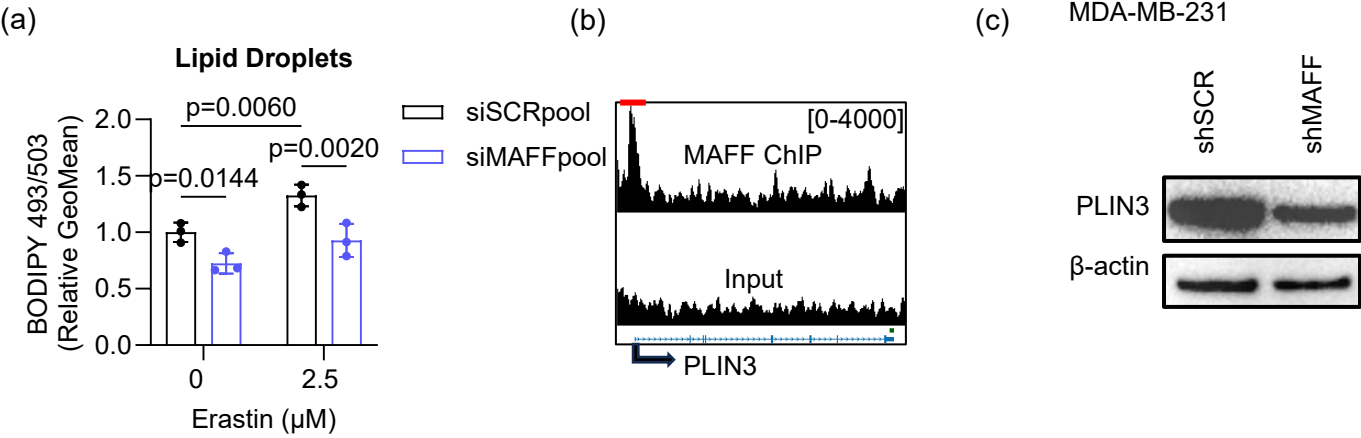

Fig. S5.

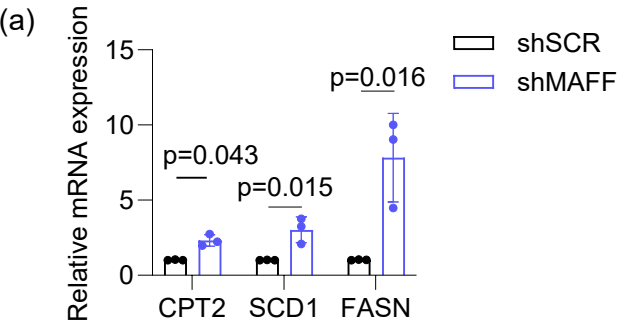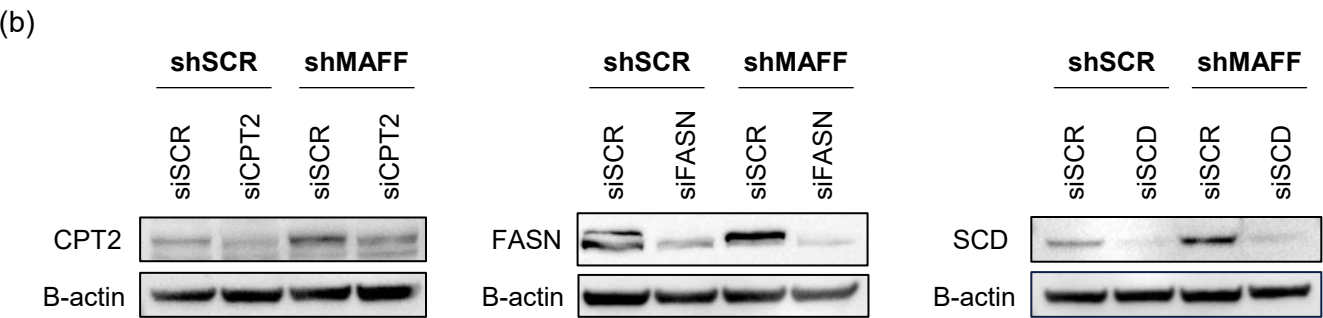

Fig. S6.

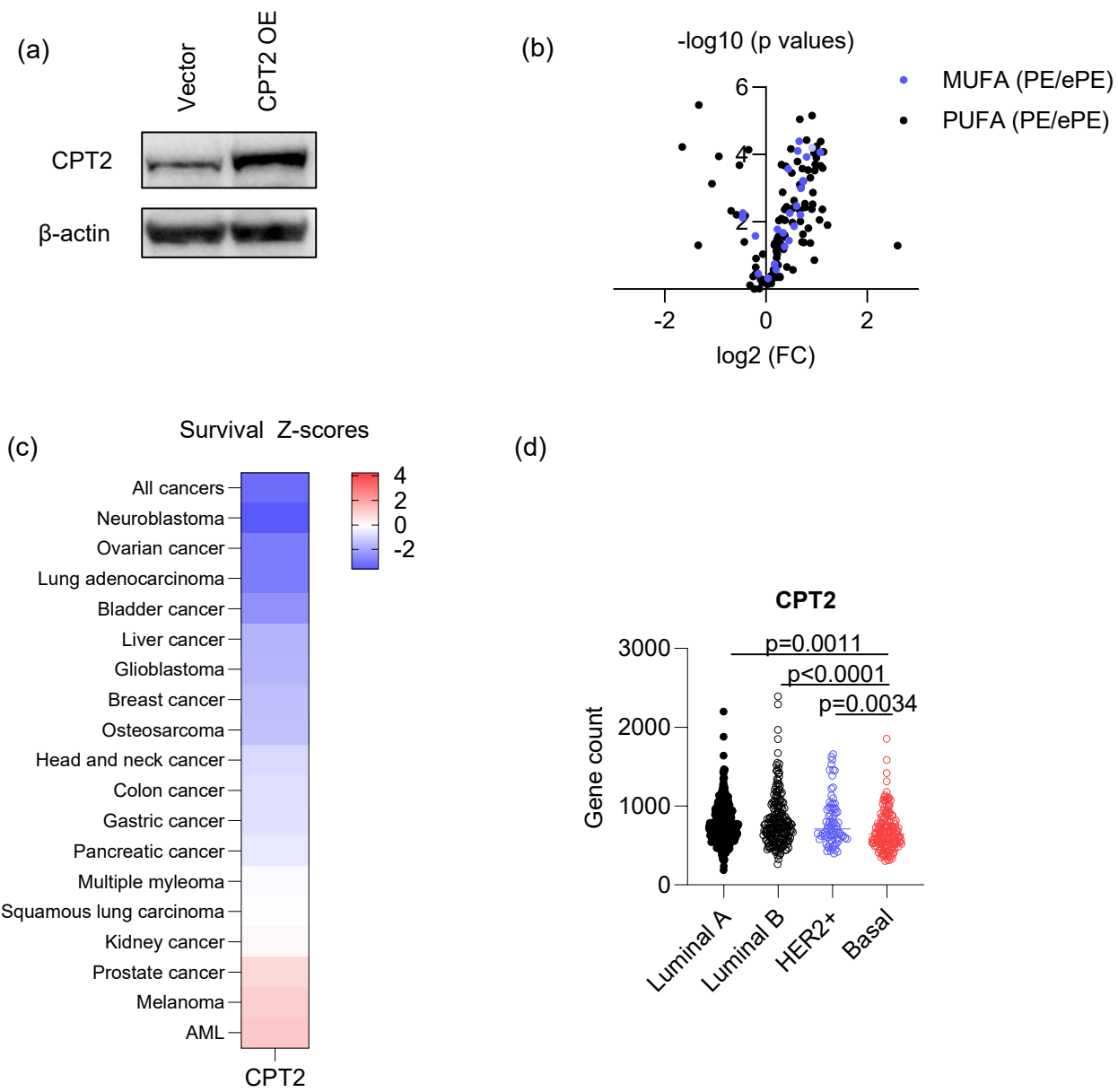

Supplement: Supplementary file 2 — Supplementary Figures [file 41419_2026_8885_MOESM2_ESM.pdf]

Fig. 3f

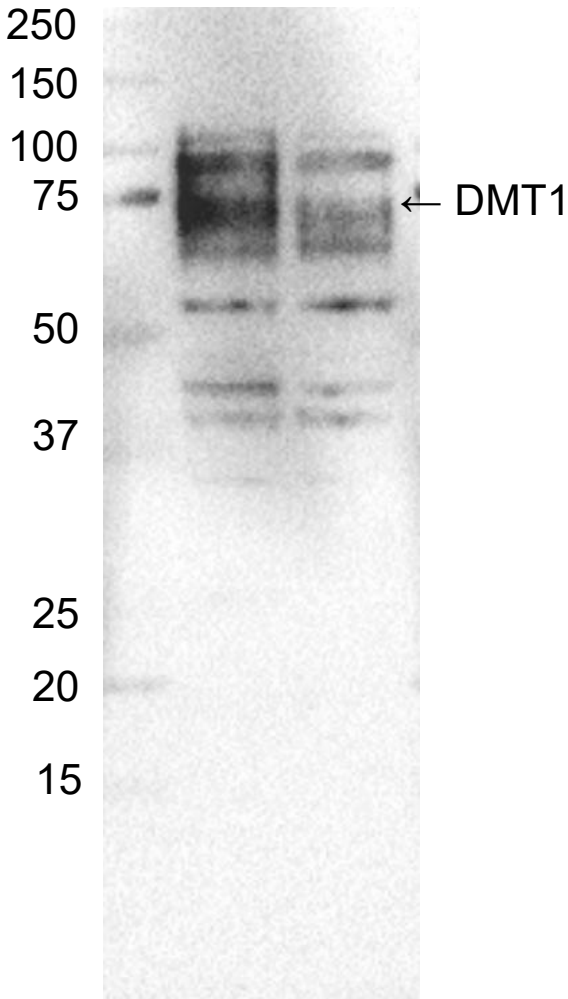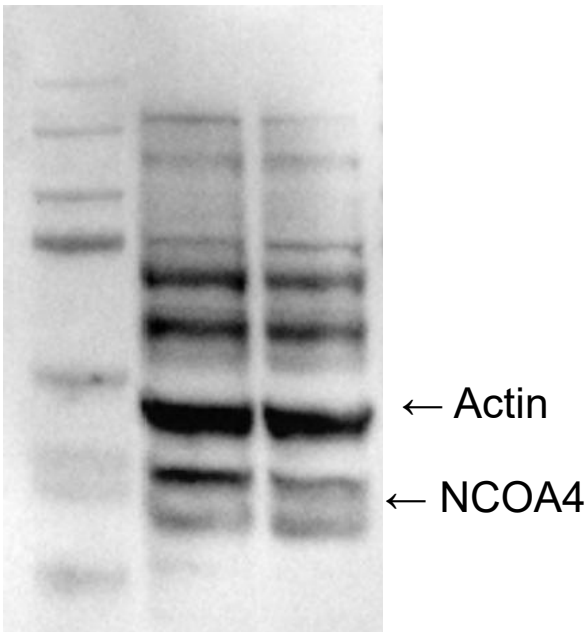

Fig. 5b

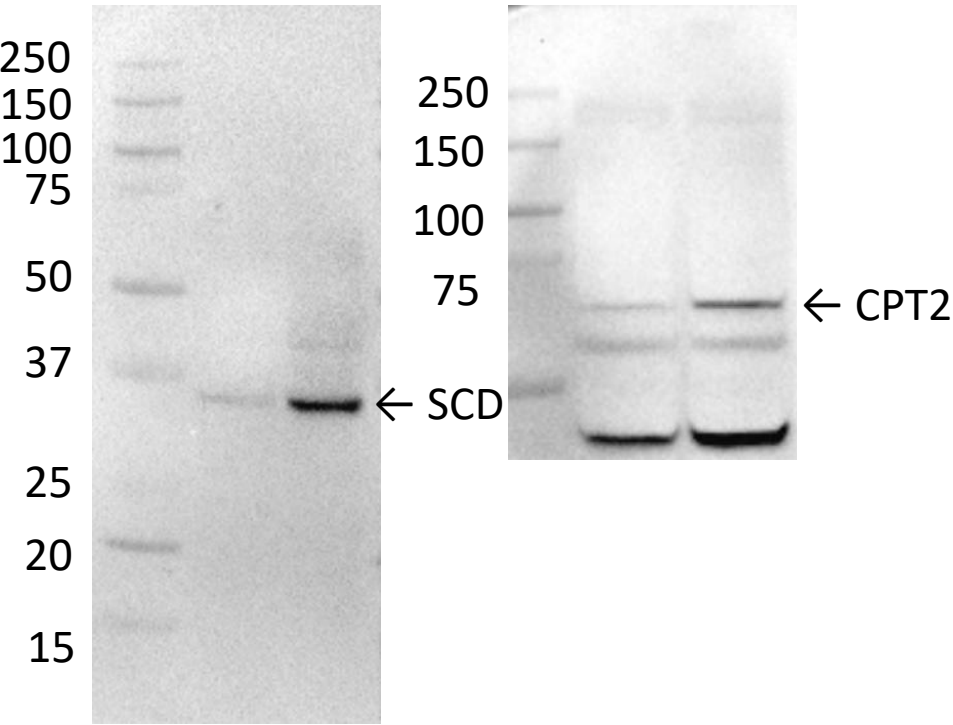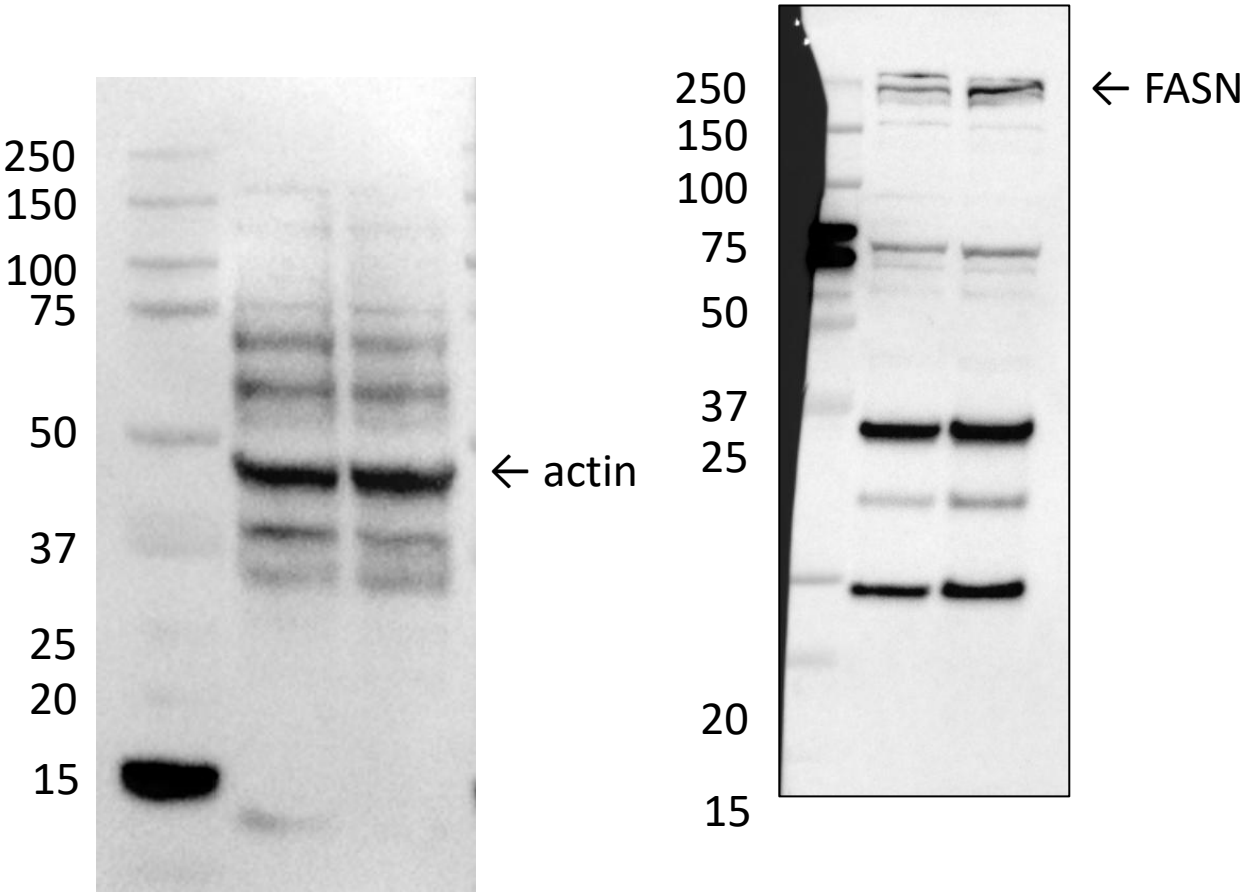

Fig. S1a

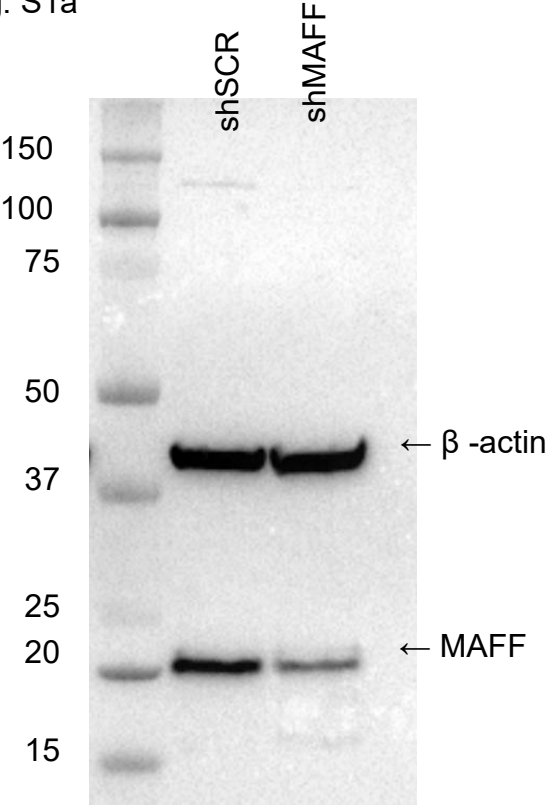

Fig. S2d

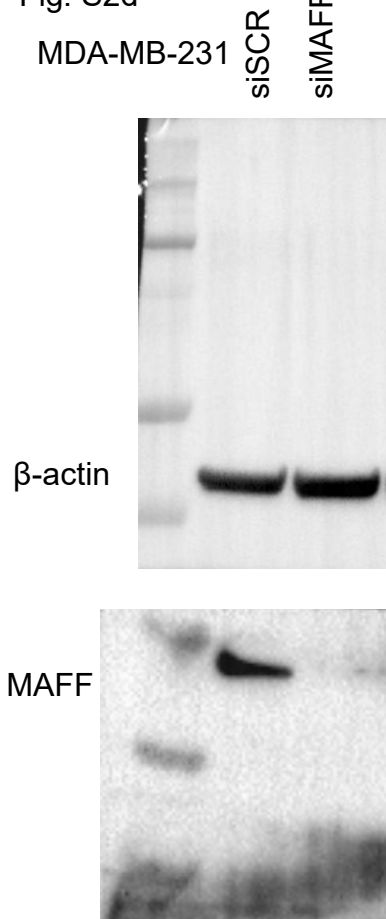

Fig. S2h

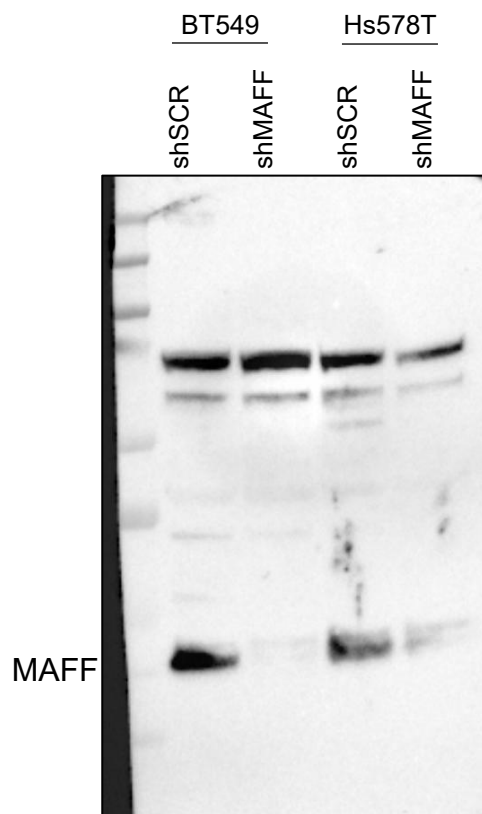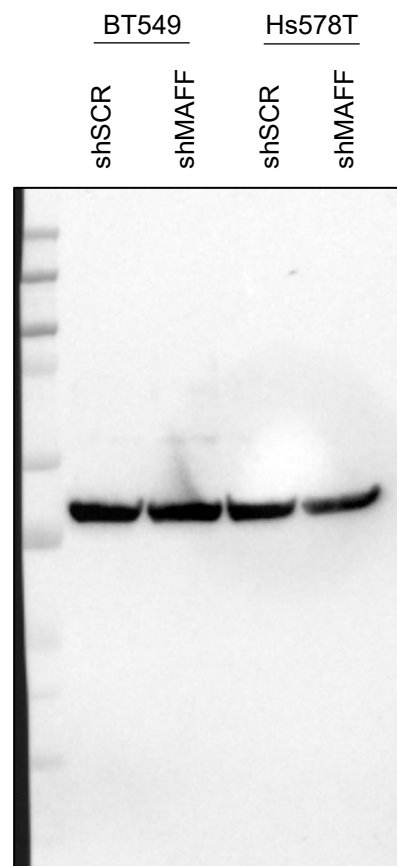

Fig. S2k

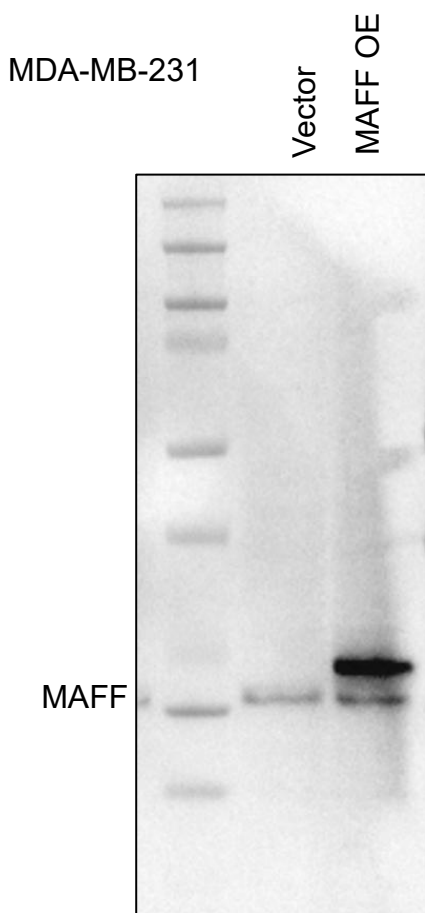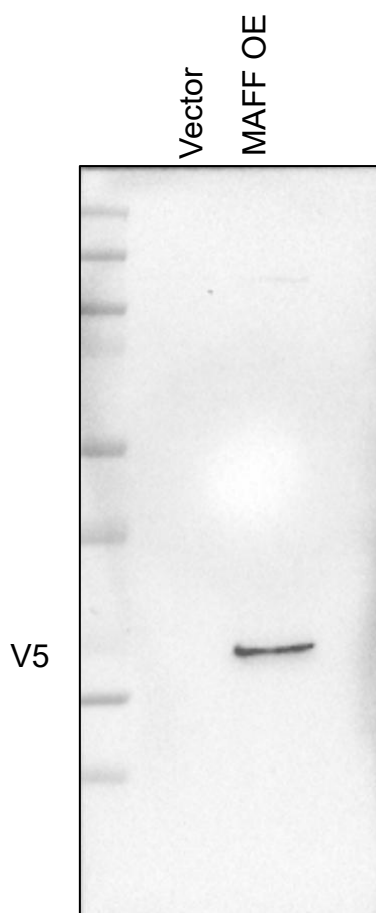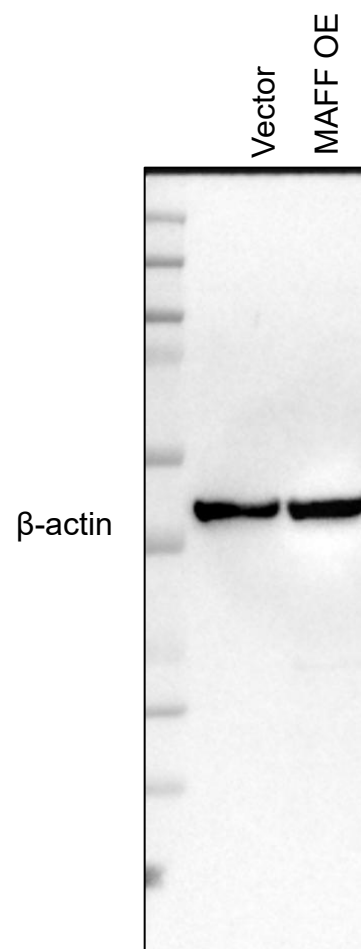

Fig. S3c

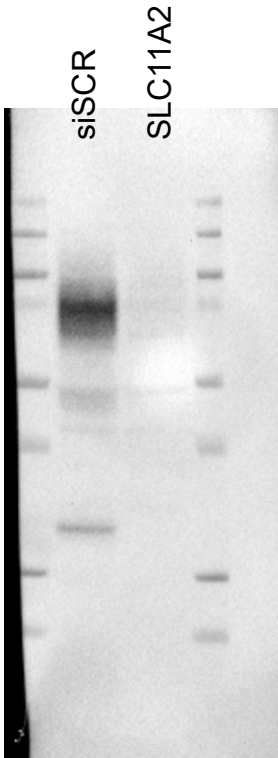

←DMT1

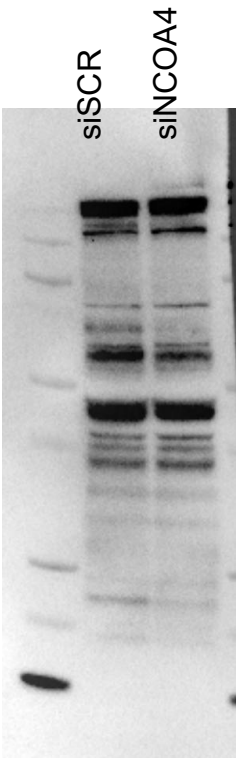

←NCOA4

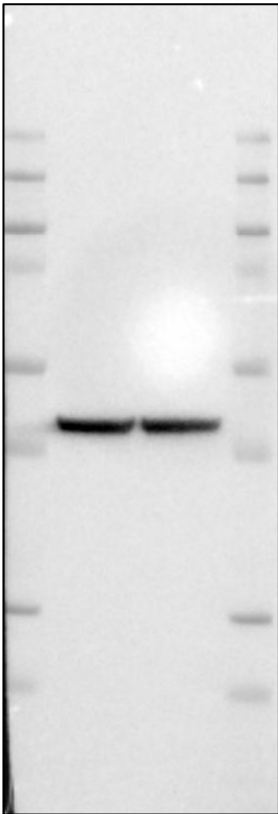

←  $\beta$  -actin

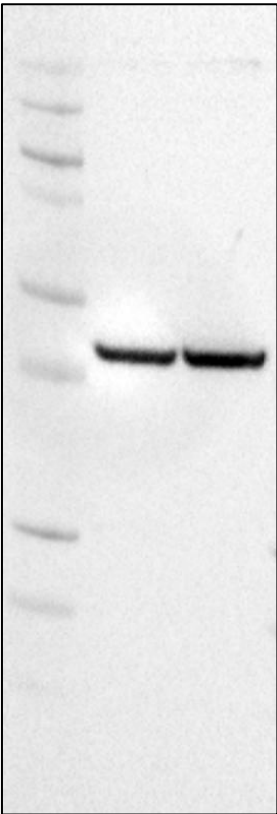

←  $\beta$  -actin

Fig. S4c

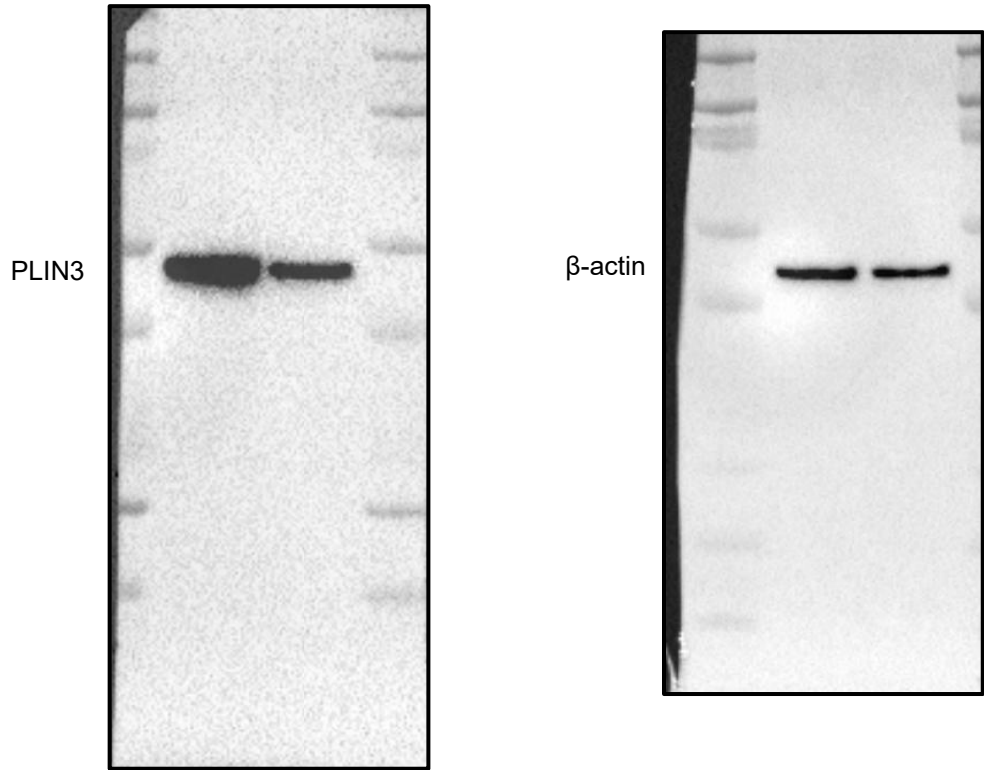

Fig. S5b

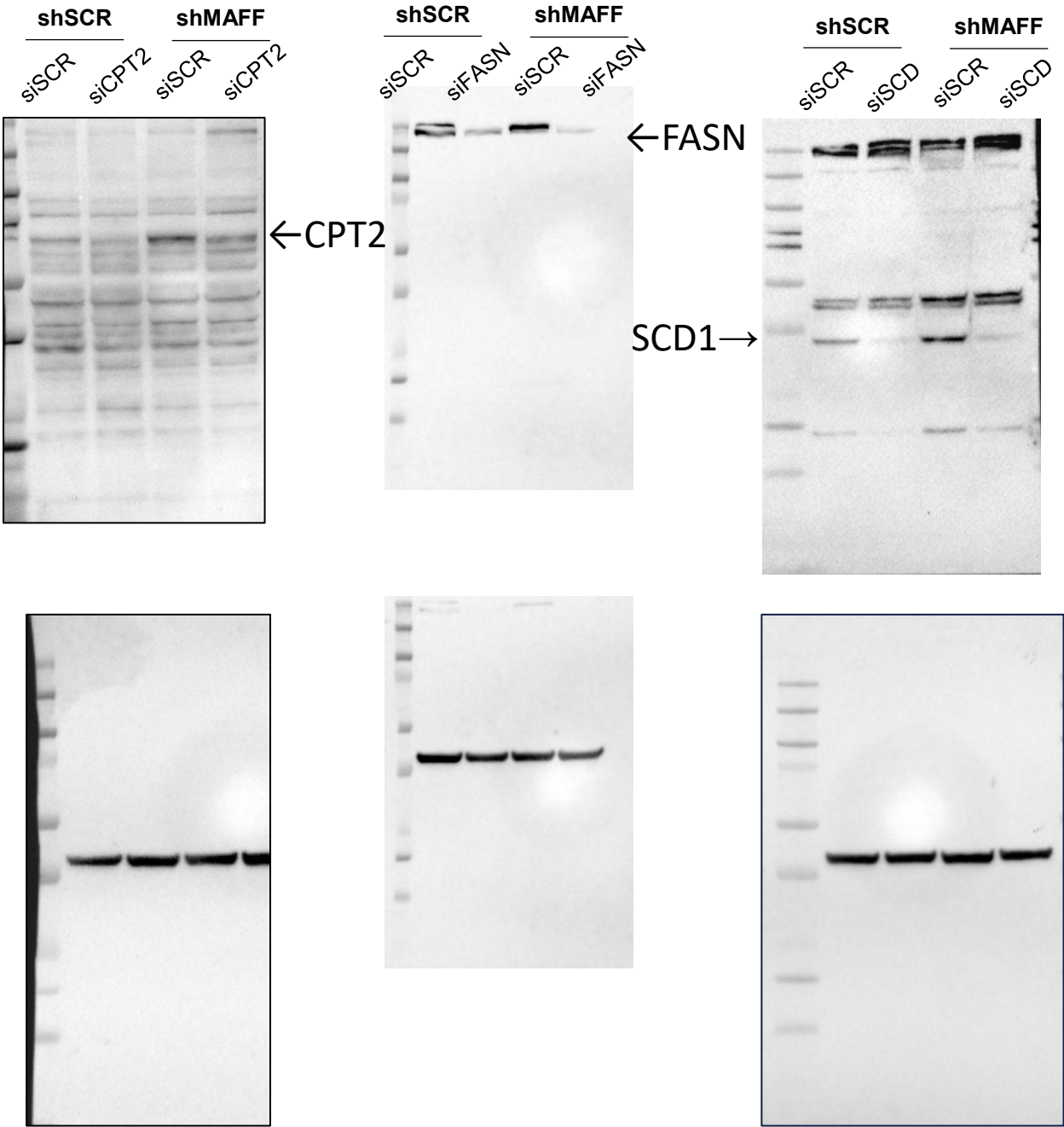

Fig. S6a

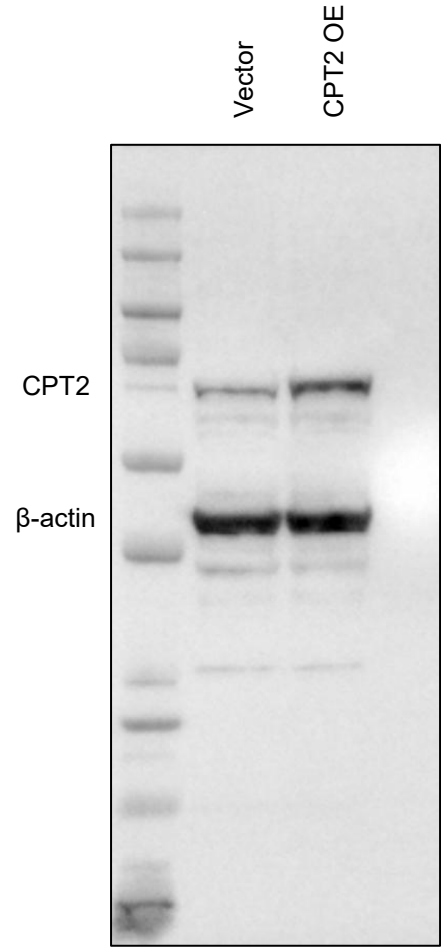

Supplement: Supplementary file 3 — Original Data [file 41419_2026_8885_MOESM3_ESM.pdf]
